# Tubb3 expression levels are sensitive to neuronal activity changes and determine microtubule growth and kinesin-mediated transport

**DOI:** 10.1007/s00018-022-04607-5

**Published:** 2022-10-29

**Authors:** Jennifer Radwitz, Torben J. Hausrat, Frank F. Heisler, Philipp C. Janiesch, Yvonne Pechmann, Michael Rübhausen, Matthias Kneussel

**Affiliations:** 1 Institute for Molecular Neurogenetics, Center for Molecular Neurobiology, ZMNH, University Medical Center Hamburg-Eppendorf, Falkenried 94, 20251 Hamburg, Germany; 2 Institut für Nanostruktur- und Festkörperphysik, Center for Free Electron Laser Science (CFEL), University of Hamburg, Luruper Chaussee 149, 22761 Hamburg, Germany

**Keywords:** Neuron, Microtubule, Tubulin, Tubb3, EB3, Motor protein, Transport, KIF5, N-Cadherin, Synapse, LTP

## Abstract

Microtubules are dynamic polymers of α/β-tubulin. They regulate cell structure, cell division, cell migration, and intracellular transport. However, functional contributions of individual tubulin isotypes are incompletely understood. The neuron-specific β-tubulin Tubb3 displays highest expression around early postnatal periods characterized by exuberant synaptogenesis. Although *Tubb3* mutations are associated with neuronal disease, including abnormal inhibitory transmission and seizure activity in patients, molecular consequences of altered Tubb3 levels are largely unknown. Likewise, it is unclear whether neuronal activity triggers *Tubb3* expression changes in neurons. In this study, we initially asked whether chemical protocols to induce long-term potentiation (cLTP) affect microtubule growth and the expression of individual tubulin isotypes. We found that growing microtubules and *Tubb3* expression are sensitive to changes in neuronal activity and asked for consequences of *Tubb3* downregulation in neurons. Our data revealed that reduced Tubb3 levels accelerated microtubule growth in axons and dendrites. Remarkably, *Tubb3* knockdown induced a specific upregulation of *Tubb4* gene expression, without changing other tubulin isotypes. We further found that Tubb3 downregulation reduces tubulin polyglutamylation, increases KIF5C motility and boosts the transport of its synaptic cargo N-Cadherin, which is known to regulate synaptogenesis and long-term potentiation. Due to the large number of tubulin isotypes, we developed and applied a computational model based on a Monte Carlo simulation to understand consequences of tubulin expression changes in silico. Together, our data suggest a feedback mechanism with neuronal activity regulating tubulin expression and consequently microtubule dynamics underlying the delivery of synaptic cargoes.

**Supplementary Information:**

The online version contains supplementary material available at 10.1007/s00018-022-04607-5.

## Introduction

The microtubule (MT) cytoskeleton represents a dynamic structure mediating a variety of cellular and subcellular functions in neurons and other cell types [1]. In addition to the regulation of cell division, cell migration and neurite outgrowth, microtubules regulate intracellular transport of organelles, vesicles, mRNAs, and signaling molecules across the cell [2]. They consist of α/β-tubulin heterodimers that form protofilaments, which dynamically assemble to form a hollow tube. Due to the nature of the dimers, representing the microtubule building blocks, microtubules contain a plus- and a minus-end. They bind a number of accessory proteins at their plus-end (+TIPs) that include EB3 [3]. The dynamic behavior of microtubule ends is characterized by the process of dynamic instability, which comprises growth, catastrophe, shrinkage, and rescue driven by GTP-hydrolysis [3]. Consistent with MTs mediating diverse functions several genes encode for alpha- and beta-tubulin. At least 8 different alpha-tubulins (Tubas) and eight different beta-tubulins (Tubbs) are expressed in the mouse. In addition, microtubules undergo posttranslational modification, such as de-tyrosination, acetylation or polyglutamylation, and are stabilized through microtubule-associated proteins (MAPs) [4–6]. Due to the many possible combinations to form α/β-tubulin heterodimers and their posttranslational modification patterns, individual microtubules are thought to have specific identities and functions [5, 7–12]. However, the complex roles of tubulin isotype functions, in particular in neurons that represent polar and excitable cell types, are just beginning to be understood. It is for instance unknown whether the expression of individual tubulin genes is regulated by neuronal activity, or vice versa, whether changes in the expression of tubulin isotypes regulates neuron-specific functions, such as synaptic plasticity, the ability of synapses to change in strength.

Plastic adaptions of neuronal transmission contribute to learning and memory in the central nervous system. For instance, long-term potentiation (LTP) induced through high-frequency synaptic stimulation [13, 14] or chemical induction [15, 16] triggers a long-lasting increase in synaptic strength. LTP also induces several pre- and postsynaptic mechanisms, such as the delivery and insertion of N-Cadherin and AMPA receptors into postsynaptic sites [17–19]. Whereas microtubule-dependent transport is critical for the activity-dependent delivery and rearrangement of synaptic factors [2, 20, 21], functional roles of individual tubulin isotypes, in particular the neuron-specific tubulins, have remained elusive.

Polymorphisms in tubulin genes of human patients lead to several neurological disorders, known as tubulinopathies [22–24]. They include Tubb3, a major tubulin isotype in brain, which is exclusively expressed in neurons and influences the dynamics of microtubules [8, 25, 26]. Knockdown of *Tubb3* gene expression or specific missense mutations were shown to influence cortical development in mice [27, 28]. It was further reported that *Tubb3* expression is upregulated in human and rat epileptic tissue [29], linking Tubb3 to neuronal activity. In *Tubb3* knockout mice, other *Tubb* genes are upregulated to compensate for the overall level of total beta-tubulin. However, specific Tubb3 functions cannot be replaced by other isotypes [30], indicating essential roles of Tubb3 in this context.

Whether Tubb3 mediates unique functions with respect to the kinesin-mediated transport of plasma membrane proteins that regulate neuronal synapses, is presently unknown. Kinesin family 5 (KIF5) motor proteins, also known as kinesin I motors, travel along microtubules to deliver a variety of subcellular cargoes across the cell [2]. In neurons, KIF5 transports vesicles containing AMPA-type glutamate receptors (AMPARs) and/or the cell adhesion molecule N-Cadherin toward synaptic sites [31, 32]. The extracellular domain of postsynaptic N-cadherins binds *in trans* to the extracellular domain of presynaptic N-cadherins, thereby spanning the synaptic cleft. N-Cadherin regulates cytoskeletal functions in association with catenins and affects voltage-dependent calcium influx [33]. It is sensitive to neuronal activity changes that induce mechanisms of synaptic plasticity [19, 34, 35]. In particular, LTP promotes the formation of N-Cadherin clusters in stimulated spines, thereby stabilizing spine structure [34]. In general, the delivery of neuronal cargoes is highly regulated by the combinatorial use of cargo adapters [2]. For instance, N-Cadherin transport through KIF5 requires the glutamate receptor-associated protein GRIP1 [32]. Other modes to regulate transport include tubulin posttranslational modifications, such as polyglutamylation. Previous studies in neurons revealed that increased polyglutamylation decreases the efficiency of neuronal transport along axons [36] or toward the synapse [37].

Here we asked whether changes in neuronal activity alter microtubule growth and/or the expression of individual tubulins. Vice versa, we aimed to understand whether the downregulation of a specific tubulin isotype alters microtubule dynamics and microtubule-dependent transport in neurons. We used the motor protein KIF5 and the LTP-sensitive cell adhesion molecule N-Cadherin (a KIF5 cargo) to monitor potential transport changes, following *Tubb3* knockdown. Our data reveal that Tubb3 expression is sensitive to neuronal activity changes, whereas *Tubb3* downregulation accelerates microtubule growth and microtubule-dependent transport of motors and cargoes.

## Materials and methods

### Antibodies

The following primary and secondary antibodies were used: mouse anti-Tubb1 (Novus; #NBP2-46245; IF 1:200); rabbit anti-Tubb2 (Abcam; #ab179512; IF 1:200); mouse anti-Tubb3 (Biolegend; #801202; IF 1:1,000; WB 1:5,000); mouse anti-Tubb4 (Novus; #NB-120-11315; IF 1:100); mouse anti-Tubb5 (Novus; #NB-120-11312; IF 1:200); mouse anti-panTuba (Sigma; #T9026; IF 1:1,000); rabbit anti-panTubb (Abcam; #ab151318; IF 1:200); rat anti-panTuba (Origene; #SM568P; IF 1:500); rabbit anti-Polyglutamate chain (PolyE) (AdipoGen; #AG-25B-0030; IF 1:1000); mouse anti-Glutamate Receptor 2 (Millipore; #MAB397; WB 1:1000); mouse anti-Homer1 (Synaptic Systems; #160011; WB 1:1000); mouse anti-GAPDH (GeneTex; #GTX28245; WB 1:5000); mouse anti-gamma-adaptin (BD Bioscience; #610386; WB 1:5,000); donkey anti-mouse HRP-conjugated (Dianova; #715-036-151; WB 1:10,000); Cy3 donkey anti-mouse IgG (Dianova; #715–165-150; IF 1:500); Cy3 donkey anti-rabbit (Dianova; #711-166-152; IF 1:500). DNA stainings were performed with: DAPI (Thermo Fischer; #D1306: 1:1,000). IRDye 680RD goat anti-mouse IgG (LI-COR, NE, USA, 926-68070, WB 1:10,000); IRDye 800CW goat anti-rabbit IgG (LI-COR, NE, USA, 926-32211, WB 1:10,000).

### Constructs

Constructs for miRNA knockdown were engineered using the BLOCK-iT_TM_ Pol II miR RNAi expression vector kit (Invitrogen; #45-1102). The following target sequences were used: miRNA-1 (CACCAGCTAGTGGAGAACACA), miRNA-2 (CAGGCCCGACAACTTTATCTT), miRNA-3 (CTCCCTTCGATTCCCTGGTCA), miRNA-4 (CGTGCGGAAAGAGTGTGAGAA), miRNA-5 (CCTAGATGTCGTGCGGAAAGA) and miRNA-scr (GGCCTTGCCGGTATATACGACAG). EB3-Tomato was cloned as Eco*RI*/Bam*HI* fragment into ptd-Tomato-N1. Tubb3 was cloned as Hind*III*/Sal*I* fragment into pAcGFP1-C3. The constructs KIF5C-tdTomato-PEX26 [37] and mRFP-N-Cadherin [32] were previously published.

### Cell culture

Neuroblastoma N2a, human embryonic kidney 293 (HEK) and COS-7 cells were cultured in DMEM supplemented with 10% fetal bovine serum and 1% penicillin–streptomycin. Cells were split every two to three days using 5% trypsin. To culture primary neurons, mouse embryos at stage E15 to E17 were used. Hippocampi were dissected in ice cold HBSS (Gibco, Thermo Scientific, #14170-088). To dissociate the cells, the hippocampi were incubated in 0.05% trypsin supplemented with EDTA at 37 °C for 5 min. The reaction was stopped with prewarmed DMEM supplemented with 10% fetal bovine serum. Cells were triturated with two fire polished Pasteur pipettes of different diameter in prewarmed HBSS. To count cell numbers, a Neubauer chamber was used. For immunofluorescent staining, 80,000 cells were seeded per 12 mm glass coverslip (24 well plate), coated with poly-l-lysine in 1 ml Neurobasal Plus Medium (Gibco, Thermo Scientific, # A35829-01). For time lapse imaging, 50,000 cells were seeded into the center of a 25 mm glass coverslip (6 well plate), coated with poly-l-lysine in 3 ml Neurobasal Plus Medium.

### Transfection

To transfect N2a cells, the ScreenFect A-plus Transfection Reagent (InCella, #S-6000-1) was used according to the manual. HEK cells were transfected using the calcium phosphate method. Primary hippocampal neurons were transfected using the calcium phosphate method or Lipofectamine, respectively. For transfection of a 12 mm coverslip (24 well plate) with the calcium phosphate method, 2 µg DNA were mixed with water to a volume of 18.75 µl and 6.25 µl CaCl_2_ (1 M) were added. This solution was mixed dropwise with 25 µl 2 × HBS (280 mM NaCl; 10 mM KCl; 1.5 mM Na_2_HPO_4_; 12 mM dextrose; 50 mM HEPES) and incubated for 10 min at room temperature (RT). For a 25 mm coverslip (6 well plate), the double amounts were used, as described. Then, two thirds of the medium were removed and the transfection mix was added, followed by an incubation period between 45 min and 2 h at 37 °C and 5% CO_2_. Afterwards, the medium containing the transfection mix was removed and the cells washed twice in HEPES buffer (10 mM HEPES; 135 mM NaCl; 5 mM KCl; 2 mM CaCl_2_; 2 mM MgCl_2_; 15 mM glucose; pH 7.4). The remaining conditioned medium was then returned to the cells. For HEK cells, the transfection mix was added to the medium and incubated overnight, then, cells were lysed or fixed. For transfecting a 25 mm coverslip (6 well plate) with Lipofectamine, 2 µg DNA was mixed with 23 µl Optimem (Opti-MEM I(1x), gibco, #31985-070). In parallel, 2 µl Lipofectamine (Lipofectamine 2000, Invitrogen, #11668-019) were mixed with 23 µl Optimem. Both solutions were mixed and incubated for 20 min. Two thirds of the neuronal medium were removed from the well and the transfection mix was added for 2 h at 37 °C and 5% CO_2_. Afterwards, the medium containing the transfection mix was removed and cells were washed twice with HEPES buffer (10 mM HEPES; 135 mM NaCl; 5 mM KCl; 2 mM CaCl_2_; 2 mM MgCl_2_; 15 mM glucose; pH 7.4). Finally, the remaining conditioned medium was returned to the well containing the cells.

### Immunocytochemistry

Cells were fixed by dipping them into PBS (137 mM NaCl; 2.7 mM KCl; 8.1 mM Na_2_HPO_4_; 1.4 mM KH_2_PO_4_) and incubating them in 4% paraformaldehyde, supplemented with 4% sucrose in water over 12 min at RT. After washing twice with PBS, cells were permeabilized in 0.25% Triton-X-100/PBS for 4 min. Then, cells were washed with PBS and blocked in 1% BSA/PBS for 60 min at RT. Afterwards, the primary antibody, diluted in 1% BSA/PBS, was applied for 60 min at RT. Following four washing steps with PBS, the secondary antibody was applied over 45 min at RT. Cells were then washed again four times with PBS and the coverslips were dipped into water prior to mounting them with Aqua-Poly/Mount (Polysciences, Inc., #18606-20) onto glass slides.

### Time-lapse video microscopy and image processing

Time lapse imaging was conducted with a spinning disc confocal microscope (Nikon ECLIPSE T*i*) at 37 °C and 5% CO_2_ using a CCD camera (Hamamatsu, EM-CCD, Digital Camera C9100). Axons and dendrites were identified by morphological characteristics. Videos were acquired with a 100 × objective (NA 1.45) and an image acquisition rate of 1 s over 3 min with the Visiview software (Visitron Systems). Fixed samples were imaged with a confocal microscope from Olympus (Olympus Fluoview FV1000) using a 60 × objective (NA 1.35) and the Fluoview software (Olympus). Analysis of the videos was conducted with an ImageJ macro.

### Chemical induction of LTP

To chemically induce long-term potentiation (cLTP) in acute hippocampal slices, the potassium ion channel blocker tetraethylammonium chloride (TEA) was used. Hippocampi of adult C57BL/6 mice were dissected and cut into 400 µm thick slices. They were kept for one hour in artificial cerebral spinal fluid (ACSF), containing 125 mM NaCl, 25 mM NaHCO_3_, 25 mM glucose, 2.5 mM KCl, 1.25 mM NaH_2_PO_4_, 1 mM MgCl_2_ and 2 mM CaCl_2_, saturated with 95% O_2_ and 5% CO_2_. Control slices were kept in ACSF only. All slices for cLTP induction were incubated for 10 min in TEA-ACSF, containing 110 mM NaCl, 25 mM NaHCO_3_, 15 mM glucose, 2.5 mM KCl, 1.25 mM NaH_2_PO_4_, 1 mM MgCl_2_, 2 mM CaCl_2_ and 25 mM TEA, saturated with 95% O_2_ and 5% CO_2_. Slices were then transferred into ACSF without TEA. A first group was just dipped (10 min group), a second group incubated for another 10 min (20 min group) and a third group incubated for another 20 min (30 min group) (compare with Fig. 6). Slices were then homogenized in lysis buffer (1% Triton-X-100; protease inhibitor complete 1x, PhosSTOP 1x; in PBS) and centrifuged at 1000×*g* at 4 °C for 10 min. 10 µl of the supernatant were used to determine the total protein concentration with the Pierce BCA Protein Assay Kit (Thermo Scientific, REF: 23227). The rest of the supernatant was mixed with 1 × SDS sample buffer (4 × stock: 250 mM Tris (pH 6.8); 40% glycerin; 8% SDS; 20% beta-mercaptoethanol; 0.008% bromophenol blue; in water) and boiled at 95 °C for 6 min. Samples were then loaded to SDS gels and processed for western blotting. To induce cLTP in dissociated mouse hippocampal neurons, neurons transfected at DIV8-9 were subjected to cLTP induction 3 days post transfection. cLTP was induced by treatment with 50 µM Forskolin, 100 nM Rolipram and 100 µM Picrotoxin [38, 39] (Tocris, Wiesbaden-Nordenstadt, Germany) for 10 min in Ringer solution without Mg^2+^ (125 mM NaCl, 2,5 mM KCl, 3 mM CaCl^2^, 33 mM d-Glucose, 25 mM HEPES, pH 7.3) (all Sigma, Steinheim, Germany) at 37 °C in a humidified incubator with 5% CO_2_. Thereafter, neurons were washed once with warm PBS and for recovery from cLTP induction, neurons were incubated in Ringer solution containing Mg^2+^ (125 mM NaCl, 2,5 mM KCl, 2 mM CaCl_2_, 33 mM d-Glucose, 1 mM MgCl_2_, 25 mM HEPES, pH 7.3). Following 10 min of recovery, neurons were subjected to live imaging as described in the time-lapse video microscopy and image processing section.

### Statistical analysis

Statistical analysis was conducted with SigmaPlot (version 14.0). First, data were tested for a normal distribution with the Shapiro–Wilk test. For normally distributed datasets, equal variance was tested with the Brown–Forsythe test. For two samples of normal distributed data, a Student’s *t* test was used for equal variances and a Welch’s *t* test for unequal variances. For three or more samples of normal distributed data, a one-way ANOVA was used with the Dunnett’s Method for multiple comparisons. If data were not normally distributed, the Mann–Whitney Rank Sum test was used for two samples and the Kruskal–Wallis one-way ANOVA on ranks with Dunn’s Method for multiple comparisons for three or more samples. Data were plotted as mean ± SEM or as box-plot. The box-plot displays the median as line, the mean as square and the box from the first quartile to the third quartile. Whiskers indicate the 1.5 interquartile range.

### Microtubule growth model

To simulate microtubule growth velocity, a two-dimensional computational Monte Carlo model was developed that incorporates three different tubulin dimer types as building blocks. Each rectangle represented one alpha- and beta-tubulin dimer, which formed the protofilaments marked by numbers 1 to 13 (Fig. 5a, right). Periodic boundaries mirrored protofilament 1 next to protofilament 13 and vice versa, to generate a continued grid. Bonds between dimers along one protofilament were characterized by longitudinal bond energies. Bonds between dimers of neighboring protofilaments were characterized by lateral bond energies. Protofilament 1 and 13 were shifted by 1.5 dimers to represent the MT seam. Accordingly, each dimer had 0, 0.5, 1, 1.5 or 2 lateral neighbors. Since we focused on microtubule growth velocities, the effects of catastrophe, shrinkage, rescue and GTP hydrolysis were neglected. During the growing phase of a microtubule, dimers stochastically bound to or dissociated from the protofilament tips, respectively. If more dimers were bound than were dissociated, the microtubule increased in length. Binding and unbinding were characterized by the bimolecular on-rate constant *k*_on_ (µM^−1^ s^−1^) and the unimolecular off-rate constant *k*_off_ (s^−1^).

They were related by the equilibrium constant *K* (µM^−1^):

1 $$K=\frac{{k}_{\mathrm{on}}}{{k}_{\mathrm{off}}}.$$

The bimolecular on-rate constant was multiplied with the tubulin concentration to give a pseudo first order on-rate constant *k*_on_ (s^−1^). The standard Gibbs free energy change was described by:

2 $$\Delta G^\circ =-\mathrm{RT ln}\left(K\right),$$

with *R* being the universal gas constant and *T* the absolute temperature in kelvin. It was assumed, that each dimer that binds to a protofilament, forms one longitudinal bond changing the free energy by ∆*G*_long_. The energy changes for dimer immobilization and conformational change were included into ∆*G*_long_ to simplify the parameter setting. For each lateral neighbor, the energy ∆*G*_lat_ was added, leading to a change in the free energy upon dimer binding of:

3 $$\Delta G=\Delta {G}_{\mathrm{long}}+x\Delta {G}_{\mathrm{lat}},$$

with *x* being the number of lateral neighbors. Therefore, with a given *k*_on_ and combining Eqs. 1, 2 and 3, *k*_*o*ff_ was calculated as:

4 $${k}_{\mathrm{off}}=\frac{{k}_{\mathrm{on}}}{\mathrm{exp}(-\frac{\Delta {G}_{\mathrm{long}}+x\Delta {G}_{\mathrm{lat}}}{RT})}.$$

### Simulation procedure

To perform the simulation, the following possible events were considered. Each protofilament either bound a type one, type two or type three dimer, reflecting individual combinations of tubulin isotypes. To consider sterical hindrance of existing lateral neighbors at the potential binding site, an on-rate penalty of 2 (*k*_on_/2) was introduced for one lateral neighbor and a penalty of 10 (*k*_on_/10) for two lateral neighbors [40]. In addition, each dimer of the grid was able to dissociate. As shown in Eq. (4), the number of lateral neighbors determined the quantity of lateral binding energies and, therefore influenced *k*_off_. If a dimer dissociated from a position inside the grid, all upper dimers of this protofilament also dissociated and all lateral energies of these dimers were added. To enable dissociation from the beginning of the simulation, protofilaments were given a start-length of 10 dimers, each. Dimer types were chosen within a Monte Carlo simulation randomly to yield a uniform distribution of dimers. In the second step, the execution time *t* (s) for each event was calculated according to the literature [41].

5 $${t}_{i}=\frac{-\mathrm{ln}({N}_{i})}{{k}_{i}},$$

with *i* being the index of the possible event, *N* being a uniformly distributed random number between 0 and 1, and *k* being the rate constant (s^−1^) of the event.

In a third step, the event with the shortest execution time was implemented and its time was added to the total amount of time. To model velocities, as observed experimentally, steps one to three were repeated 5,000 times. Simulation parameters were *k*_on_, ∆*G*_long_(1), ∆*G*_long_(2), ∆*G*_long_(3), ∆*G*_lat_(1), ∆*G*_lat_(2), ∆*G*_lat_(3) as well as the tubulin dimer concentrations C1, C2 and C3 (parameters and final values are summarized in Fig. 6b). The final output was the time of growth and the length of the protofilaments, from which the growth velocity of the microtubule tip could be calculated by:

6 $$\mathrm{velocity}= \frac{\text{mean length of protofilaments}}{\text{total elapsed time}}.$$

### Parameter determination

Tubulin heterodimers consist of one α-tubulin and one β-tubulin. In the present study, we focused on β-tubulin isotypes, therefore α-tubulin isotypes were not differentiated. To determine the parameters, the model was adjusted to match experimental EB3 imaging data. Accordingly, dimer type 1 was defined to represent Tubb3-containing dimers and dimer type 2 to represent Tubb4-containing dimers. Since free energy changes are additive, dimer type 3 was defined to represent all other β-tubulin isotype-containing dimers (Fig. 5a). With the aim to predict longitudinal and lateral energies of Tubb3 and Tubb4 (∆*G*_long_(Tubb3), ∆*G*_lat_(Tubb3), ∆*G*_long_(Tubb4), ∆*G*_lat_(Tubb4)), published values for the on-rate constant *k*_on_ = 30 (µM^−1^ s^−1^) and the total tubulin concentration *C* = 7 µM were adapted from a study by Castle and colleagues [42]. Longitudinal and lateral energy values (∆*G*_long_(other Tubbs) =  − 7 RT and ∆*G*_lat_(other Tubbs) = − 4 RT) for type 3 dimers were estimated based on references [40–43]. From RNA data in the literature [7, 44], it was estimated that Tubb3 represents 14%, Tubb4 28% and the remaining Tubbs 58% of soluble tubulin in the neuronal cytoplasm. Using these values and a tubulin concentration of 7 µM, individual tubulin dimer concentrations were calculated for control conditions (C(Tubb3) = 0.98 µM, C(Tubb4) = 1.96 µM and C(other Tubbs) = 4.06 µM). For *Tubb3* knockdown conditions, individual dimer concentrations were determined using our experimental results from this study. Following *Tubb3* knockdown, *Tubb3* expression levels were set to 43%, representing an average value of different experiments in this study and *Tubb4* expression levels were set to 133%. Based on this, molarities of individual Tubbs were calculated (C(Tubb3) = 0.42 µM, C(Tubb4) = 2.61 µM and C(other Tubbs) = 3.97 µM). To determine longitudinal and lateral energies of Tubb3- and Tubb4-containing dimers, the program was executed with longitudinal energies ranging from − 3 RT to − 9 RT in 0.5 RT steps and lateral energies ranging from -1 RT to the respective longitudinal value. This procedure was justified, since longitudinal energies have to be smaller than lateral energies to polymerize stable MTs [45].

For each energy parameter set (∆*G*_long_(Tubb3), ∆*G*_lat_(Tubb3), ∆*G*_long_(Tubb4), ∆*G*_lat_(Tubb4)), microtubule growth velocities were calculated under control or *Tubb3* knockdown conditions, respectively. Since plotting 5-dimensional data were not feasible, results were collected and processed using Excel (Microsoft), as described below. To display the results, energy values for Tubb3 and Tubb4 were plotted separately in contour graphs (Fig. 5b–e). To plot Tubb3 energies, Tubb4 energy values were fixed to final result values (∆*G*_long_(Tubb4) = − 7.5 RT, ∆*G*_lat_(Tubb4) = − 6 RT). Likewise, the same procedure was applied for Tubb4 plots (∆*G*_long_(Tubb3) = − 5 RT, ∆*G*_lat_(Tubb3) = − 3 RT). Gray contour lines were displayed to mark experimental velocities (Control: 0.167 µm/s; *Tubb3* KD: 0.201 µm/s). From the original results parameter sets that generated velocities in the range of: scrambled control: 0.167 ± 0.006 µm/s; mi-RNA4-mediated *Tubb3* knockdown: 0.201 ± 0.008 µm/s, were selected. Parameter sets that coincided for control and knockdown conditions were filtered, resulting in 88 parameter sets that fulfilled the requirements (Table S1). To narrow their number, the program was executed with these parameter sets, calculating 100 velocities under control and *Tubb3* knockdown concentrations, each. Since the velocity standard error of mean (SEM) for 100 calculations turned out to be 0.002 µm/s, energy parameter sets generating similar velocities (scrambled control: 0.167 ± 0.002 µm/s; mi-RNA4-mediated *Tubb3* knockdown: 0.201 ± 0.002 µm/s) were selected. This procedure resulted in four parameter sets that fulfilled the requirements (Table 1).

**Table 1:** Energy parameter settings after refinement

G_long_(Tubb3) in RT	G_lat_(Tubb3) in RT	G_long_(Tubb4) in RT	G_lat_(Tubb4) in RT	Velocity control in μm/s	Velocity knockdown in μm/s
− 5	− 3	− 7.5	− 6	0.167	0.200
− 4	− 3	− 7.5	− 6.5	0.169	0.201
− 7	− 1	− 5	− 8	0.166	0.203
− 6	− 1.5	− 6	− 7.5	0.166	0.201

100 microtubule growth velocity values were calculated for each of the filtered energy parameter sets, using control or *Tubb3* knockdown concentrations, respectively. The velocity standard error of the mean (SEM), representing 100 calculations was 0.002 µm/s. Energy parameter sets that generated velocities in this range (scrambled control: 0.167 ± 0.002 µm/s; mi-RNA4-mediated *Tubb3* knockdown: 0.201 ± 0.002 µm/s) were selected. Parameter sets that coincided for control or knockdown conditions were filtered. This procedure resulted in four individual parameter settings that fulfilled the requirements

## Results

### Increase in microtubule growth and tubulin expression following cLTP induction

**Fig. 1:**
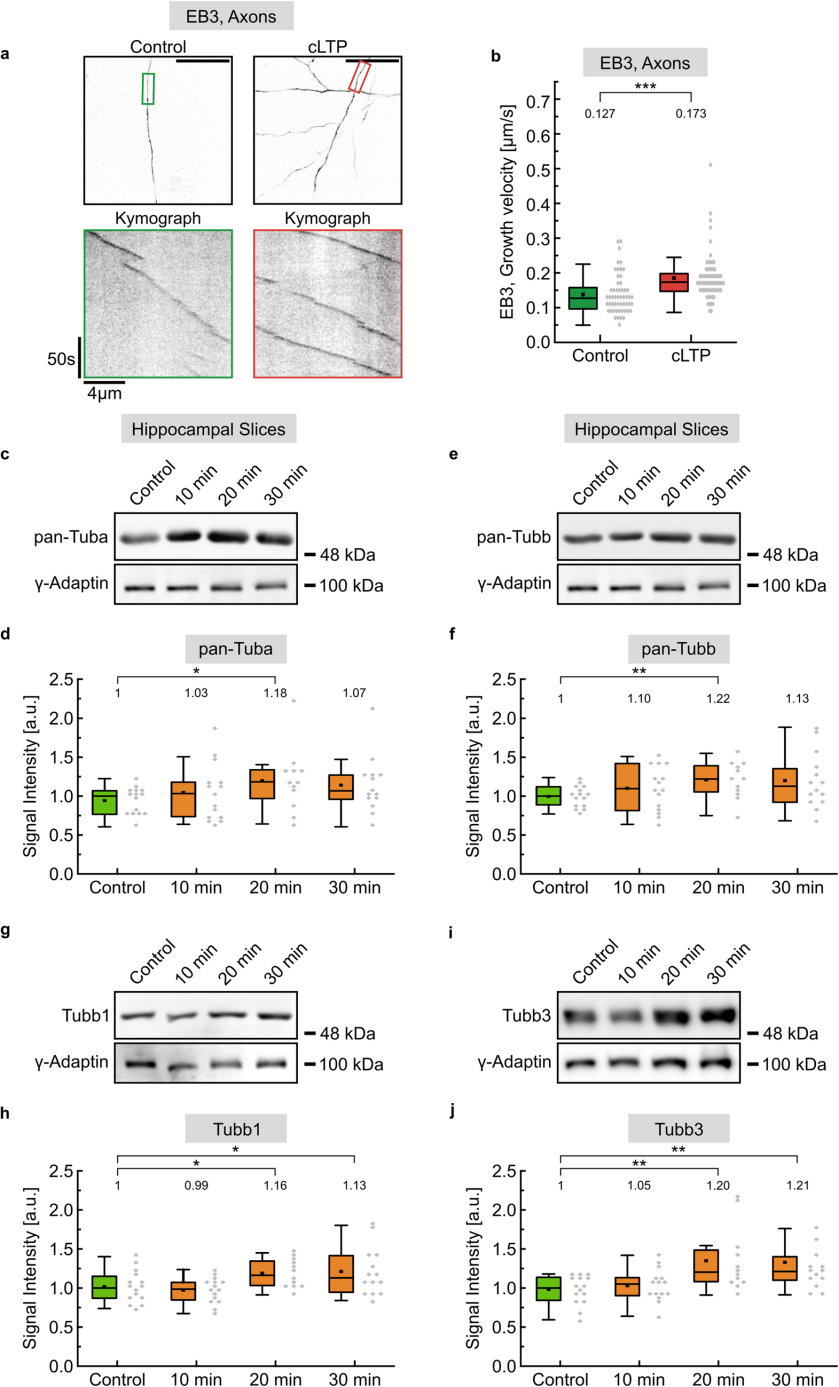
Microtubule growth and tubulin expression following cLTP induction. **a** Live cell imaging of DIV 11/12 cultured hippocampal neurons transfected with EB3-Tomato and exposed to chemical induction of LTP. To induce cLTP, neurons transfected at DIV8-9 were subjected to cLTP induction 3 days post transfection. cLTP was induced by treatment with 50 µM Forskolin, 100 nM Rolipram and 100 µM Picrotoxin for 10 min in Ringer solution without Mg^2+^, following 10 min of recovery in Ringer solution containing Mg^2+^. Control cells were kept in Ringer solution containing Mg^2+^. Image acquisition: 1 frame/s over 3 min. Scale bar, 20 µm. EB3 comets in axonal segments in colored boxes of overview images (top panel) are visualized by kymographs (bottom panel). **b** Quantification of a, depicting median values (*N* = 3 experiments). ***p < 0.001. Each data point represents one EB3 comet; control (*n* = 54), cLTP (*n* = 70). For graphical representation of the distribution, data are binned with a bin size of 0.02 µm/s. Statistics: Mann–Whitney Rank Sum Test. **c**–**j** Quantification of tubulin isotype expression in acute hippocampal slices, following chemical induction of LTP. Control slices were kept in ACSF. cLTP slices were incubated in ACSF with TEA for 10 min and further incubated in pure ACSF over 0, 10 or 20 min. (gamma-Adaptin: loading control; *N* = 3 experiments, each). Representative western blots and the corresponding quantification of (**c**, **d**) pan-Tuba, (**e**, **f**) pan-Tubb, (**g**, **h**) Tubb1 and (**i**, **j**) Tubb3. Depicted are median values. Black dots show the mean value. For graphical representation of the distribution, data are binned with a bin size of 0.05 a.u. Statistics: One way ANOVA followed by Student's t-test or Mann–Whitney Rank Sum Test. *p < 0.05, **p < 0.01

Neurons are excitable cells that adapt intracellular transport processes in response to activity changes [17, 20, 46]. They are characterized by a dynamic microtubule cytoskeleton consisting of different tubulin isotypes [3, 8, 47, 48], however it is barely understood whether activity changes induce changes in tubulin expression and whether a differential use of specific tubulin isotypes alters microtubule and/or transport function. In an initial experiment, we applied established chemical protocols to induce long-term potentiation (cLTP) to trigger a long-lasting increase in synaptic strength in cultured hippocampal neurons [38, 39, 49]. Using a fusion protein of the microtubule + TIP factor EB3 (EB3-Tomato), known to label growing microtubules [50, 51], we found that increased synaptic strength leads to a significant increase in microtubule growth velocities, as compared to control conditions (Fig. 1a, b and Figure S1a, b). Following induction of cLTP in acute hippocampal slices over different time periods (10–30 min), we further observed significantly increased expression levels for total alpha- and beta-tubulin in the range of about 20%, detected by western blotting with pan-Tuba or pan-Tubb-specific antibodies, respectively. Significantly increased gene expression was detectable at 20 min following cLTP induction and declined afterwards (Fig. 1c–f). In a subsequent isotype-specific analysis, we confirmed the upregulation of tubulins, in particular of Tubb1 and Tubb3, which displayed significantly increased expression levels at 20 and 30 min after cLTP induction (Fig. 1g–j). In contrast, the tubulin isotypes Tubb2, Tubb4 and Tubb5 remained unaltered under these conditions (Figure S2a–f), indicating that a long-lasting increase in synaptic activity [49] does not generally affect tubulin expression. Likewise, within this early phase of cLTP, which is known to be independent of the synthesis of neurotransmitter receptors [52], but is based on receptor rearrangement from endocytic reserve pools [17, 53], the expression of the AMPA receptor subunit GluA2 or the postsynaptic protein Homer remained unaltered (Figure S2g–l). Together, we conclude that individual tubulin expression levels and microtubule growth rates are sensitive to neuronal activity changes.

### Neuronal microtubules grow faster and longer following *Tubb3* downregulation

**Fig. 2:**
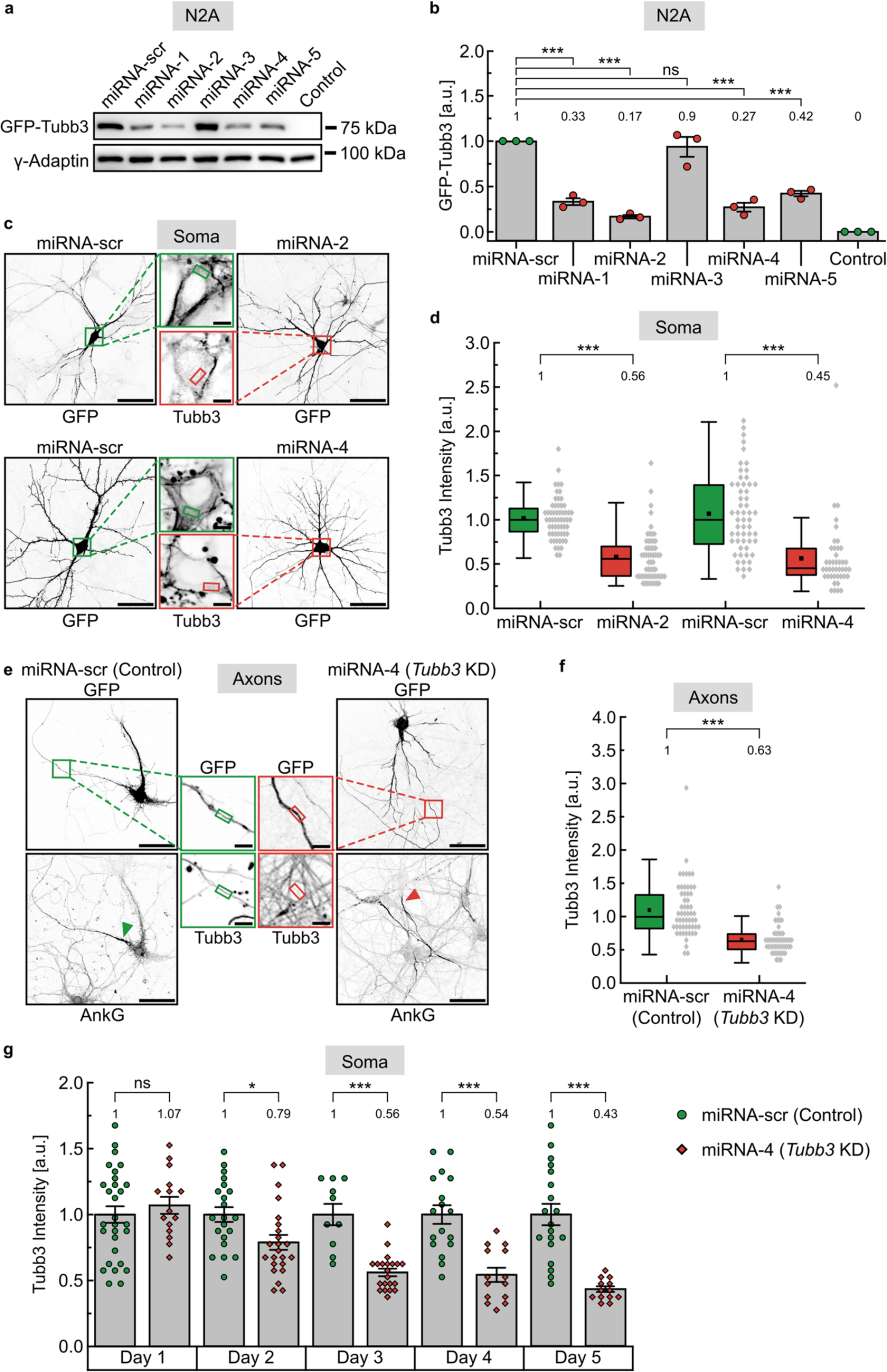
Knockdown of *Tubb3* gene expression. **a** Western blot analysis of GFP-Tubb3 expression in N2a cells, following cotransfection of miRNA constructs (miRNA-scr for scrambled control and miRNA-1 to 5) to assess their *Tubb3* knockdown capacity. Gamma-Adaptin served as loading control. Control conditions were equally treated with transfection reagent, but without DNA. **b** Quantification of a (*N* = 3 experiments). Statistics: one-way ANOVA, followed by Dunnett's Method for multiple comparisons versus control group. mean ± SEM. **c** Cultured hippocampal neurons transfected with miRNA-scr, miRNA-2, or miRNA-4, respectively. Scale bar, 50 µm. Somata in boxed regions are enlarged in the middle (green: control, red: knockdown). Tubb3 intensities were measured at DIV12 inside the soma, indicated by the small colored boxes. DAPI staining of the nucleus (not shown) was used to verify that Tubb3 intensity measurements were performed in the cytoplasm. Scale bar, 5 µm. **d** Quantification of **c**, depicting median values (*N* = 3 experiments). Each data point represents one cell; miRNA-scr (*n* = 59), miRNA-2 (*n* = 57), miRNA-scr (*n* = 56), miRNA-4 (*n* = 42). Statistics: Mann–Whitney Rank Sum Test. For graphical representation of the distribution, data are binned with a bin size of 0.08 a.u. **e** Cultured hippocampal neurons transfected with miRNA-scr (Control) or miRNA-4 (*Tubb3* KD), respectively. Scale bar, 50 µm. Axons were identified by an Ankyrin-G staining of the axon initial segment (AnkG). Axonal segments in boxed regions are enlarged in the middle (green: control, red: knockdown). Tubb3 intensities were measured at DIV12 indicated by the small colored boxes. Scale bar, 5 µm. **f** Quantification of **e**, depicting median values (*N* = 3 experiments). Each data point represents one cell; miRNA-scr (Control) (*n* = 56), miRNA-4 (*Tubb3* KD) (*n* = 42). Statistics: Mann–Whitney Rank Sum Test. For graphical representation of the distribution, data are binned with a bin size of 0.1 a.u. **g** Time course experiment as shown in c (intensities measured in the soma) over five consecutive days. *p < 0.05; ***p < 0.001; ns not significant. ± SEM values are shown

Since Tubb3 represents a neuron-specific tubulin isotype [23], we aimed to downregulate *Tubb3* gene expression to assess functional consequences. Initially, we tested five different *Tubb3* mi-RNA knockdown constructs (miRNA-1 to 5), compared to a scrambled control (miRNA-scr), following transfection in neuroblastoma N2a cells. Western blot analysis revealed that four individual constructs reduced GFP-Tubb3 significantly, leading to remaining protein levels in the range of 17 to 42% (Fig. 2a, b). Out of these candidates, we chose miRNA-2 and miRNA-4 to further test their knockdown capacity in mouse cultured hippocampal neurons. Based on the much lower transfection efficiency of neurons, immunocytochemistry turned out to be a suitable assay to assess Tubb3 protein levels in the cell soma (Fig. 2c). Quantitative analysis of fluorescent intensities in multiple regions of interest (small rectangles) confirmed that miRNA-2 and miRNA-4 significantly reduce endogenous Tubb3, leading to Tubb3 intensities of 56% or 45% compared to scrambled control levels, respectively (Fig. 2c, d, Fig. S3). Likewise, reduced Tubb3 expression was found in neuronal axons that were detected by Ankyrin G immunostaining of their initial segment (Fig. 2e, f). A neuronal time course experiment revealed that *Tubb3* gene expression gradually decreased to about 50% from day three onwards (Fig. 2g). These levels equal those of heterozygous human patients, carrying *Tubb3* loss-of-function mutations [23]. For further analysis, we therefore focused on construct miRNA-4 (hereafter referred to as *Tubb3* KD) at three days after transfection to investigate functional consequences of reduced Tubb3 levels.

**Fig. 3:**
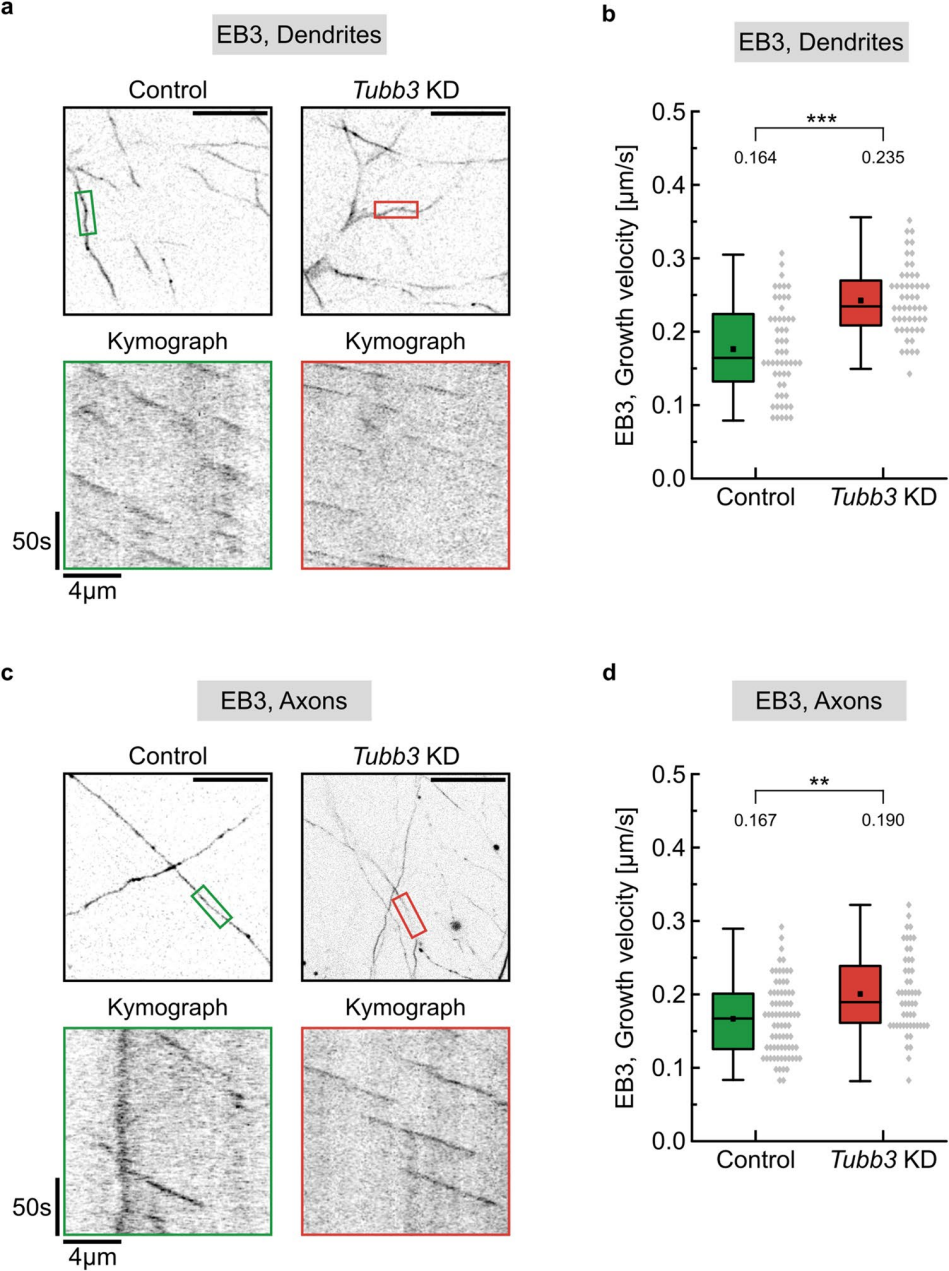
EB3 imaging to detect microtubule growth following *Tubb3* knockdown. **a** Live cell imaging of DIV 12 cultured hippocampal neurons cotransfected with EB3-Tomato and control or *Tubb3* knockdown constructs in dendrites. Image acquisition: 1 frame/s over 3 min. Scale bar, 20 µm. EB3 comets in dendritic segments in colored boxes of overview images (top panel) are visualized by kymographs (bottom panel). **b** Quantification of a, depicting median values (*N* = 3 experiments). ***p < 0.001. Each data point represents one EB3 comet; control (*n* = 55), knockdown (*n* = 52). For graphical representation of the distribution, data are binned with a bin size of 0.015 µm/s. Statistics: Mann–Whitney Rank Sum Test. **c** Live cell imaging of DIV 12 cultured hippocampal neurons cotransfected with EB3-Tomato and control or *Tubb3* knockdown constructs in axons. For details compare with a. **d** Quantification of c, depicting median values (*N* = 3 experiments). **p < 0.01. Each data point represents one EB3 comet; control (*n* = 75), knockdown (*n* = 52). For graphical representation of the distribution, data are binned with a bin size of 0.015 µm/s. Statistics: Mann–Whitney Rank Sum Test

First, we combined reduced *Tubb3* gene expression (*Tubb3* KD) with EB3-imaging in hippocampal neurons. Under control conditions (Control), we observed microtubule growth velocities with a median value of 0.164 µm/s in neuronal dendrites. Upon reduction of Tubb3 levels (*Tubb3* KD), microtubules grew significantly faster (*p* < 0.001) with a median value of 0.235 µm/s (Fig. 3a, b). They also grew about 46% longer on average (Control: 4.69 µm; *Tubb3* KD: 6.85 µm), whereas their growth duration remained equal (Figure S4a, b). Likewise, in axons, microtubules grew faster (*p* < 0.01), when Tubb3 was reduced to about half of control levels (Control: 0.167 µm/s; *Tubb3* KD: 0.190 µm/s) (Figs. 3c, d and S4c, d). To exclude off-target effects, increased EB3 growth velocities were confirmed following Tubb3 downregulation through an independent miRNA sequence (Figure S5). These data show, that the knockdown of Tubb3 modulates microtubule growth and suggest that the concentration of individual tubulin isotypes matters with respect to the dynamics of the growing microtubule cytoskeleton.

### *Tubb3* knockdown induces an upregulation of *Tubb4* gene expression

**Fig. 4:**
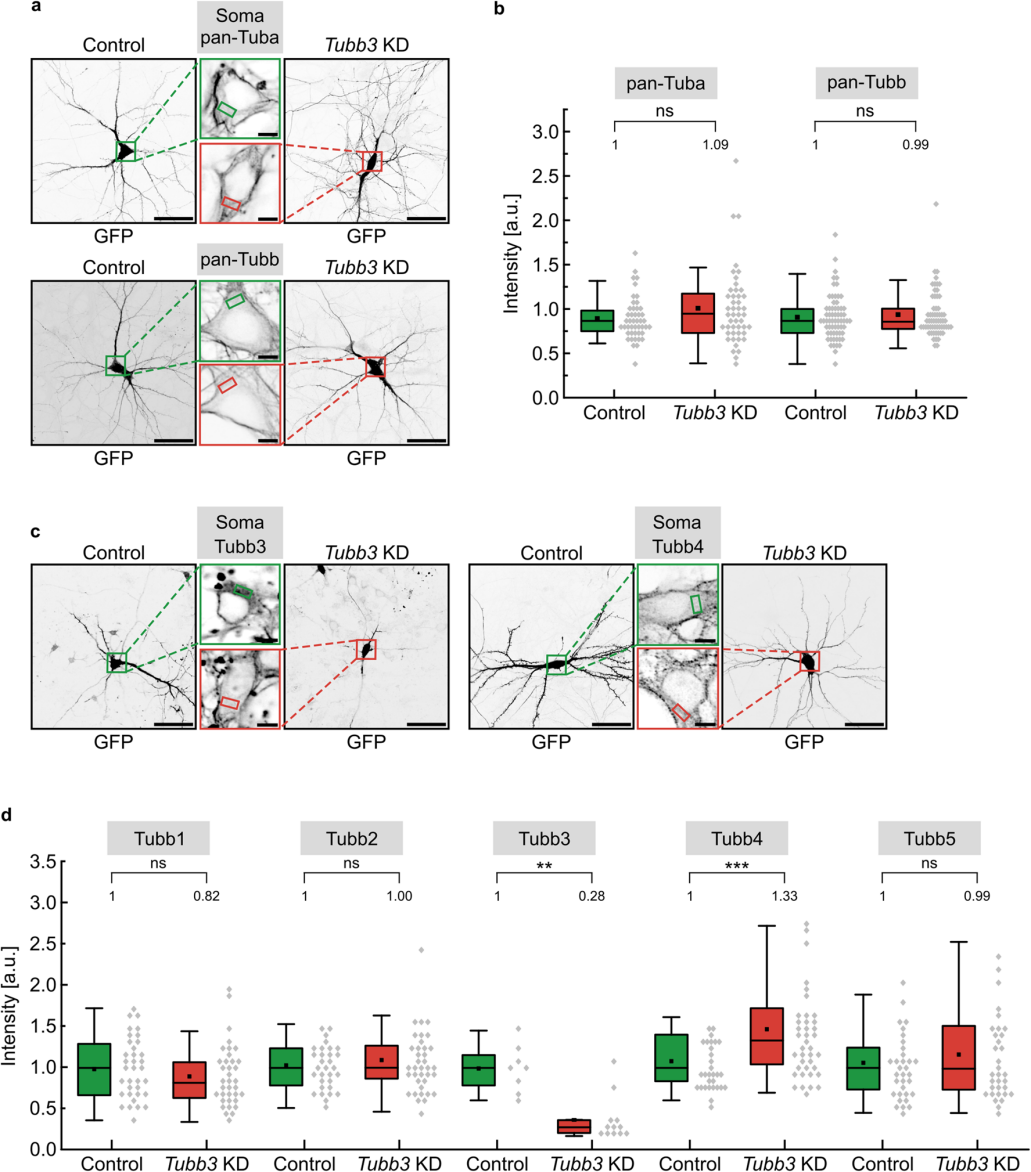
Upregulation of *Tubb4* gene expression following *Tubb3* knockdown. **a** Cultured hippocampal neurons (DIV12) transfected with control or *Tubb3* knockdown constructs. Upper: Detection with a pan-alpha antibody to probe for total-tubulin. Lower: Detection with a pan-beta tubulin antibody. Scale bar, 50 µm. Middle panels: Enlargements of cell somata boxed regions. Tubulin intensities were measured inside the soma, indicated by the small colored boxes. DAPI staining of the nucleus (not shown) was used to verify that Tubb3 intensity measurements were performed in the cytoplasm. Scale bar, 5 µm. **b** Quantification of a, depicting median values (*N* = 3 experiments). The intensity levels of pan-Tuba and pan-Tubb show no significant (n.s.) decrease, following transfection of *Tubb3* KD construct, as compared to control, set to 1. Each data point in the graph represents one cell (panTuba: Control (*n* = 46), *Tubb3* KD (*n* = 47). panTubb: Control (*n* = 64), *Tubb3* KD (*n* = 60). For graphical representation, data were binned with a bin size of 0.08 a.u. Statistics: Mann–Whitney Rank Sum Test. **c** Cultured hippocampal neurons (DIV12) transfected with control or *Tubb3* knockdown constructs. Left: Detection with a specific Tubb3 antibody. Right: Detection with a specific Tubb4 antibody. Scale bar, 50 µm. Middle panels: Enlargements of cell somata boxed regions. Tubulin intensities were measured inside the soma, indicated by the small colored boxes. DAPI staining of the nucleus (not shown) was used to verify that tubulin intensity measurements were performed in the cytoplasm. Scale bar, 5 µm. **d** Quantification of **c** and data shown in Figure S7, depicting median values (*N* = 3 experiments). **p < 0.01, ***p < 0.001, ns not significant. Each data point in the graph represents one cell. Tubb1: Control (*n* = 35), *Tubb3* KD (*n* = 36), Tubb2: Control (*n* = 31), *Tubb3* KD (*n* = 37), Tubb3: Control (*n* = 8), *Tubb3* KD (*n* = 12), Tubb4: Control (*n* = 30), *Tubb3* KD (*n* = 39), Tubb5: Control (*n* = 33), *Tubb3* KD (*n* = 33). For graphical representation, data were binned with a bin size of 0.08 a.u. Statistics: Mann–Whitney Rank Sum Test

To assess whether reduced neuronal Tubb3 alters the gene expression of other alpha- or beta-tubulin isotypes in general, we analyzed their protein levels using pan-tubulin antibodies. At least eight different alpha-tubulin and eight different beta-tubulin isotypes are expressed in the mouse [7]. Following *Tubb3* knockdown, total alpha-tubulin (pan-Tuba) levels turned out to be unchanged (Control: 1.00 a.u.; *Tubb3* KD: 1.09 a.u.) (Fig. 4a, b). Likewise, total beta-tubulin (pan-Tubb) levels were not significantly altered using a general beta-tubulin antibody (Control: 1.00 a.u.; *Tubb3* KD: 0.99 a.u.) (Figs. 4a, b, S6). However, an isotype-specific analysis revealed that *Tubb3* knockdown conditions (Control: 1.00 a.u.; *Tubb3* KD: 0.28 a.u.; Fig. 4c, d) induce a significant upregulation of the Tubb4 isotype (Control: 1.00 a.u; *Tubb3* KD: 1.33 a.u.; Figs. 4c, d, S6) in hippocampal neurons, whereas the gene expression levels of Tubb1, Tubb2 and Tubb5 remained unchanged (Figs. 4d, S7). These data raise the possibility that *Tubb4* upregulation might contribute to the acceleration of microtubule growth, as observed through EB3 imaging (compare with Fig. 3).

### Establishment of a computational model to predict microtubule growth velocities

**Fig. 5:**
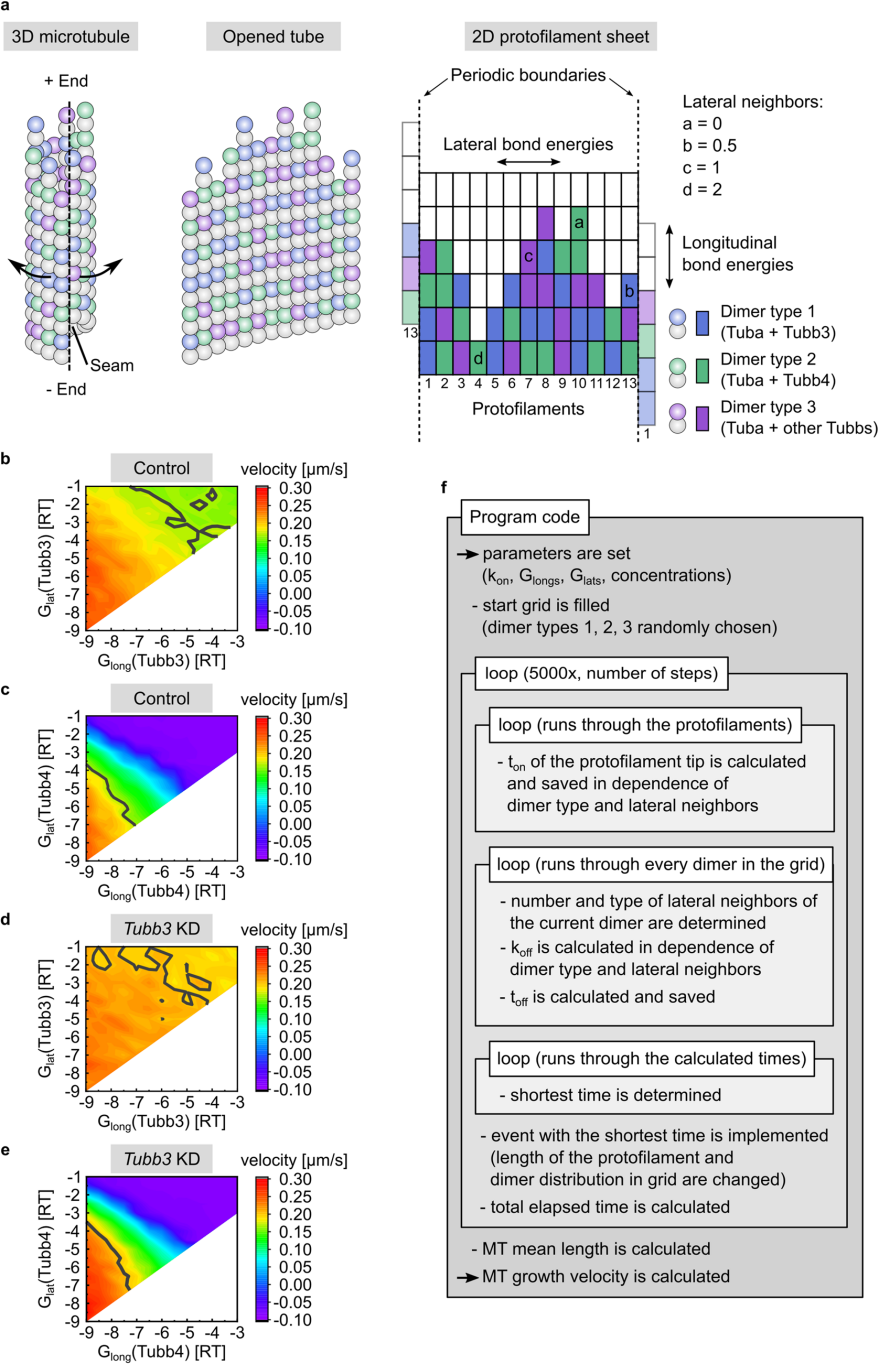
Computational model to simulate microtubule growth velocities in silico. **a** A three-dimensional microtubule tube (left) is displayed in an open conformation (middle). The dashed line represents the seam. In the flat two-dimensional protofilament sheet (right), α/β-tubulin heterodimers are simplified as single-colored rectangles which mark dimer types 1, 2 or 3. Dimer binding forms 13 protofilaments that assemble into a hollow tube, the microtubule. Periodic boundaries mirror protofilament 1 next to protofilament 13 and vice versa, so that a continues grid is generated. Longitudinal and lateral bond energies are depicted as vertical and horizontal arrows, respectively. Dimers a, b, c, and d differ in the number of lateral neighbors. Illustration of 1.5 lateral neighbors is not shown. **b**–**e** Contour graphs of longitudinal and lateral energy values of Tubb3 and Tubb4 dimers. Velocity values are color coded as indicated to the right. Gray contour lines mark experimentally measured velocities (control: 0.167 µm/s; *Tubb3* knockdown: 0.201 µm/s). In stable microtubules, longitudinal energies are smaller than lateral energies [39], therefore no velocity values were calculated for the parameter sets at the bottom right (white areas). **b** Tubb3 energy values under control conditions. **c** Tubb4 energy values under control conditions. **d** Tubb3 energy values under *Tubb3* knockdown condition. **e** Tubb4 energy values under *Tubb3* knockdown condition. **f** Scheme of the program code

**Fig. 6:**
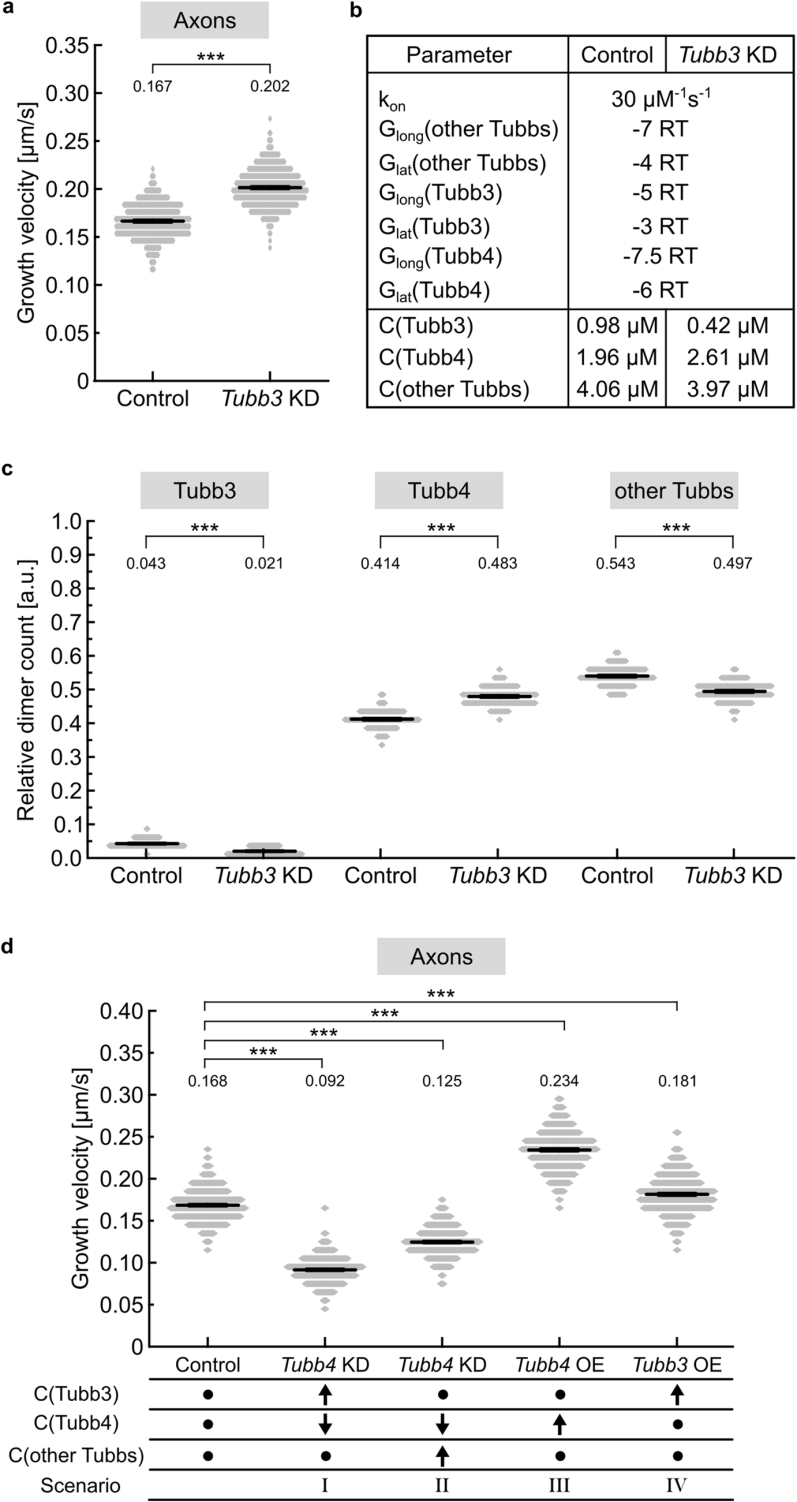
Application of the computational model. **a** The program was executed with control and *Tubb3* knockdown conditions (400 runs, each) using the parameter values listed in **b**. Data equal the experimental data of Fig. 3c, d. Mean values ± SEM are depicted. For graphical representation of the distribution, data in are binned with a bin size of 0.0075 µm/s. Statistics: Student’s *t* test. **b** Values resulting from the parameter determination process. **c** The program was executed for different conditions, as indicated (100 runs each). The number of Tubb3-containing dimers, Tubb4-containing and remaining Tubb isotype-containing dimers were counted and normalized by the total dimer count under control or *Tubb3* KD conditions, respectively. *** *p* < 0.001. Mean values ± SEM are depicted. For graphical representation of the distribution, data are binned with a bin size of 0.025 a.u. Statistics: Student’s *t* test. **d** The program was executed for different conditions, as indicated (400 runs, each), compared to control conditions. Scenario I was modeled with concentrations of C(Tubb3) = 2.1 µM, C(Tubb4) = 0.84 µM, and C(other Tubbs) = 4.06 µM. Changes compared to control concentrations are indicated by black arrows. Scenario II was modeled with concentrations of C(Tubb3) = 0.98 µM, C(Tubb4) = 0.84 µM, and C(other Tubbs) = 5.18 µM. Scenario III was modeled with concentrations of C(Tubb3) = 0.98 µM, C(Tubb4) = 2.61 µM, and C(other Tubbs) = 4.06 µM. Scenario IV was modeled with concentrations of C(Tubb3) = 2.1 µM, C(Tubb4) = 1.96 µM, and C(other Tubbs) = 4.06 µM. Altered parameters are indicated by black arrows, unchanged parameters (compared to control) by black dots. For graphical representation, data are binned with a bin size of 0.010 µm/s. Statistics: One way ANOVA, followed by Dunnett’s method for comparisons against control. ***p < 0.001

Due to the large number of possible tubulin isotypes in mammalian neurons and the limited access to isotype-specific antibodies, we developed a computational model to predict microtubule growth in silico. A two-dimensional approach was applied, which incorporates three different tubulin dimers (types 1–3) in a flattened protofilament sheet, consisting of alpha-/beta-tubulin dimers (colored rectangles, Fig. 5a, right). In the model, dimer type 1 represents Tubb3-containing dimers, dimer type 2 represents Tubb4-containing dimers and dimer type 3 the remaining Tubb isotype-containing dimers. To reduce complexity, we neglected catastrophe, shrinkage, rescue and GTP hydrolysis. Model parameters, constants as well as the simulation procedure are summarized in the methods section (Fig. 5a–f). To refine the system, we used 400 runs of the program, calculating microtubule growth velocities under control and *Tubb3* knockdown conditions, respectively. This led to an accuracy in the standard error of the mean (SEM) in the range of 0.001 µm/s. Under these conditions, we found that the parameter setting (∆*G*_long_(Tubb3) = − 5RT, ∆*G*_lat_(Tubb3) = − 3RT, ∆*G*_long_(Tubb4) = − 7.5RT and ∆*G*_lat_(Tubb4) = − 6RT) mimicked our experimental EB3 imaging results (Fig. 6a, b; compare with Fig. 3c, d).

After determination of suitable energy parameters (Fig. 6b) simulating the EB3 imaging experiment (Fig. 3d), we used these settings to assess the number of individual dimer types incorporated into growing microtubules. The program was applied with 100 runs per condition and the actual numbers of dimer types were normalized to the total amount of dimers. Under control conditions, 4.3% turned out to be Tubb3-containing dimers (Type 1), 41.4% Tubb4-containing dimers (Type 2) and 54.3% dimers containing other Tubbs (Type 3) (Fig. 6c, control conditions). In contrast, under conditions modeling *Tubb3* knockdown, we obtained a 50% reduction in Tubb3-containing dimers (2.1%, Type 1), as expected (Fig. 6c, Tubb3 dimers/*Tubb3* KD, compare with Fig. 2d). In parallel, Tubb4-containing dimers (Type 2) turned out to be increased to 48.3% (Fig. 6c, Tubb4 dimers/*Tubb3* KD, compare with Fig. 4c, d), while dimers-containing other Tubbs (Type 3) accounted for 49.7% (Fig. 6c, other Tubb dimers/*Tubb3* KD). These results confirm former experimental data suggesting that reduced Tubb3 levels can be compensated by increased Tubb4.

Together, the computational model was able to mimic our experimental results (Figs. 2, 3, 4). We therefore conclude that it is suitable to predict further neuronal microtubule growth values in silico.

### Modeling microtubule growth in silico

Next, we aimed to simulate other combinations of Tubb expression levels with respect to their capacity in regulating microtubule growth velocity. Initially, we modeled a scenario of increased Tubb3 expression, referred to as scenario I (high Tubb3, low Tubb4, equal other Tubbs). 400 runs of the program predicted that scenario I will lead to a significant decrease in the growth velocities of microtubules, compared to control conditions (control: 0.168 ± 0.001 µm/s; scenario I: 0.092 ± 0.001 µm/s) (Fig. 6d, left). Likewise, scenario II (equal Tubb3, low Tubb4, more other Tubbs, 400 runs) predicts a significant decrease in microtubule growth velocities (control: 0.168 ± 0.001 µm/s; scenario II: 0.125 ± 0.001 µm/s) (Fig. 6d). Consistent with this view, rising Tubb4 levels in turn are predicted to speed up microtubule growth in scenario III (equal Tubb3, high Tubb4, equal other Tubbs; control: 0.168 ± 0.001 µm/s; scenario III: 0.234 ± 0.002 µm/s) (Fig. 6d). Interestingly, this seems to be different for Tubb3. While low Tubb3 speeds up microtubule growth, increased Tubb3 expression is predicted to also accelerate microtubule growth (high Tubb3, equal Tubb4, equal other Tubbs; control: 0.168 ± 0.001 µm/s; scenario IV: 0.181 ± 0.002 µm/s) (Fig. 6d).

**Fig. 7:**
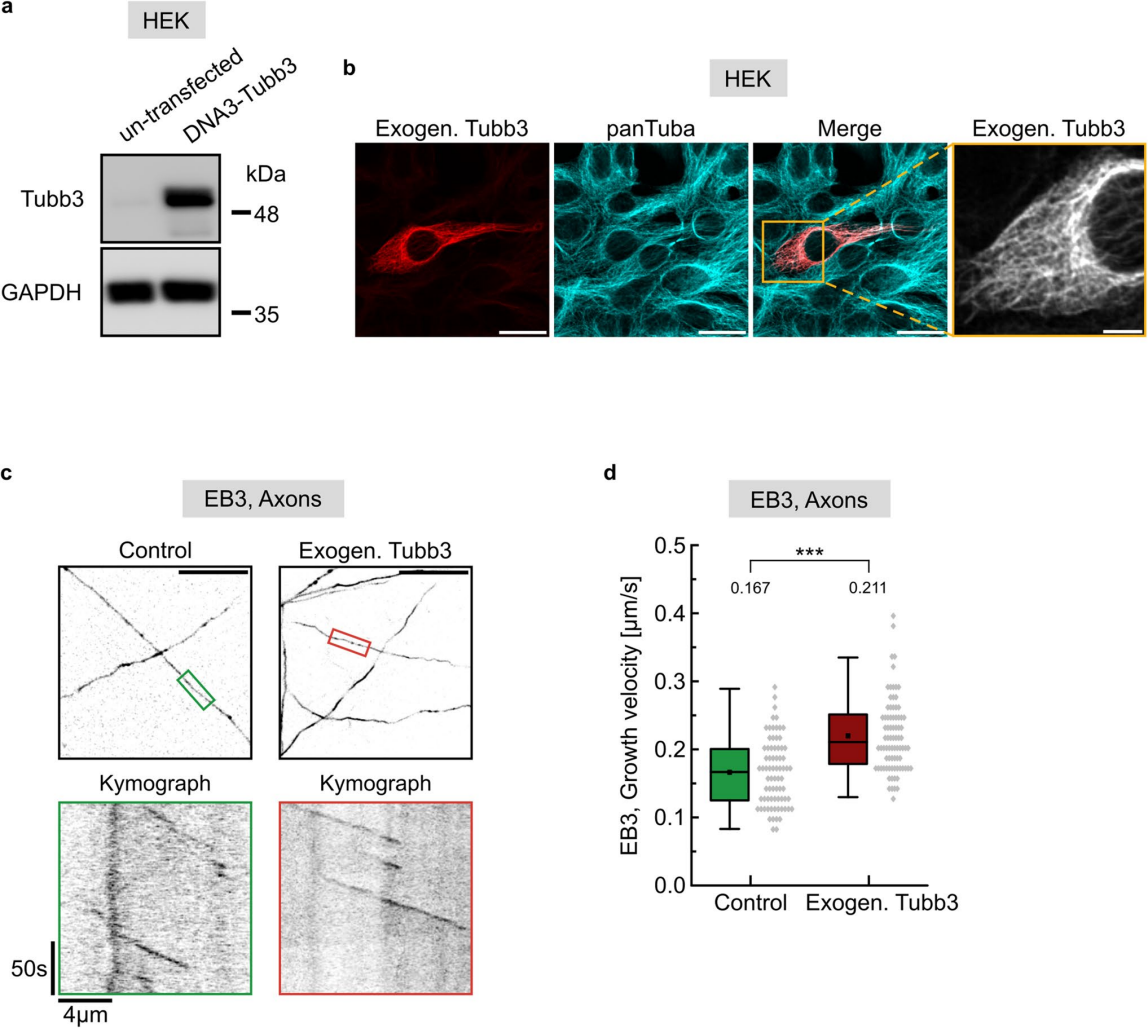
EB3 imaging to detect microtubule growth following exogenous Tubb3 expression. **a**, **b** Exogenous Tubb3 expression in HEK 293 cells. **a** Representative western blot of lysed HEK 293 cells that were untransfected or transfected with DNA3-Tubb3. After transfection, expression of Tubb3 was detectable. GAPDH was used as loading control. **b** Representative confocal images of HEK 293 cells cotransfected with DNA3-Tubb3 and GFP. 24 h later cells were fixed and immunostained for Tubb3 (exogen. Tubb3) and alpha-tubulin (panTuba). Transfected cells were identified via GFP expression (not shown). The staining illustrates the colocalization of Tubb3 with endogenous tubulin (merge). Scale bar, 20 µm. Right panel: enlargement of the cotransfected cell (yellow box) shown in the merge. Tubb3 is incorporated into filamentous microtubules. Scale bar, 5 µm. **c** Live cell imaging in axons using DIV 12 cultured hippocampal neurons cotransfected with EB3-Tomato and control or Tubb3 constructs. Image acquisition: 1 frame/s over 3 min. Scale bar, 20 µm. EB3 comets in axonal segments in colored boxes of overview images (top panel) are visualized as kymographs (bottom panel). **d** Quantification of c, depicting median values (*N* = 3 experiments). ***p < 0.001. Each data point represents one EB3 comet; control (*n* = 75), exogenous Tubb3 (*n* = 82). For graphical representation of the distribution, data are binned with a bin size of 0.015 µm/s. Statistics: Mann–Whitney Rank Sum Test

To experimentally validate this in silico prediction, we aimed to test whether the latter result (Fig. 6d, scenario IV) would be reproduceable in a neuronal context. Control transfections using a mammalian *Tubb3* expression construct induced high Tubb3 levels in HEK293 cells that lack endogenous Tubb3 expression (47) (Fig. 7a). Immunostaining with a Tubb3-specific antibody confirmed that heterologously expressed Tubb3 colocalizes with the microtubule cytoskeleton (Fig. 7b), suggesting that this approach is suitable to increase Tubb3 levels within microtubules. Finally, EB3 imaging following exogenous Tubb3 expression in hippocampal neurons, significantly increased microtubule growth velocities in axons (Fig. 7c, d), as predicted by computational modeling (compare with Fig. 6d, scenario IV).

In summary, we conclude that the expression levels of individual tubulin isotypes translate into individual functions with respect to the dynamics of microtubules. Due to the large number of alpha- and beta-tubulins and the many dimer combinations, computational modeling may be suitable to identify critical players.

### Tubb3 levels affect polyglutamylation and kinesin-mediated cargo transport along microtubules

Since dynamic microtubules represent the tracks along which motors move, we asked whether the transport of motors and cargoes might be affected following *Tubb3* knockdown. Cargo transport in neurons is highly sensitive to changes in tubulin posttranslational modifications (PTMs) [5, 54, 55]. Actually, increased polyglutamylation levels were shown to reduce the efficiency of KIF5-mediated neuronal transport, whereas decreased polyglutamylation levels increase axonal mitochondria transport [36, 37, 56]. In contrast, it is barely understood whether the concentration of specific tubulin isotypes contributes to changes in neuronal transport parameters.

**Fig. 8:**
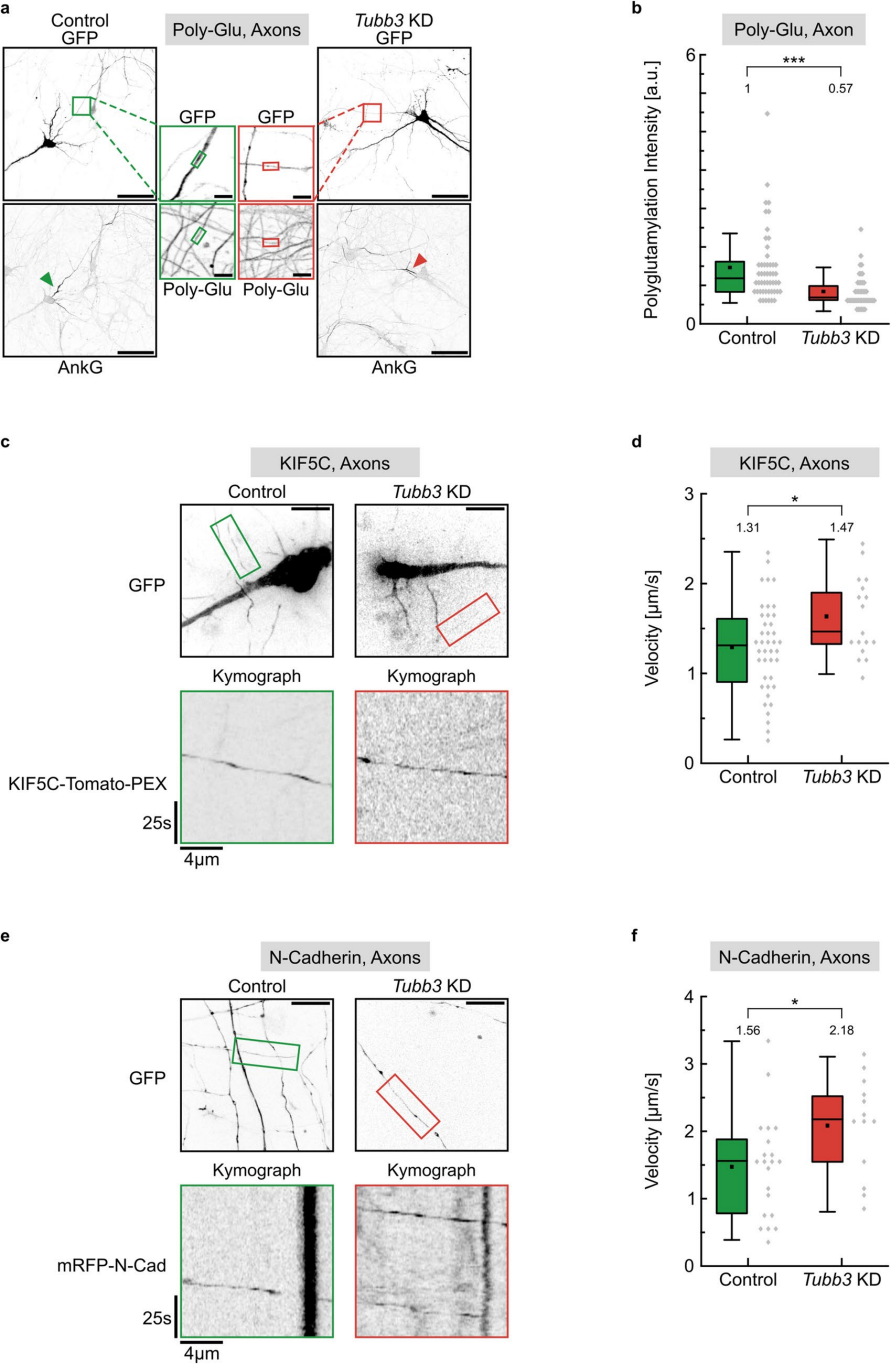
Tubulin polyglutamylation levels, KIF5C motor protein mobility, and N-Cadherin transport following *Tubb3* knockdown. **a** Cultured hippocampal neurons transfected with control or *Tubb3* KD constructs, respectively. Scale bar, 50 µm. Axons were identified by an Ankyrin-G staining of the axon initial segment (AnkG). Axonal regions in boxed regions are enlarged in the middle (green: control, red: knockdown). Polyglutamylation intensities were measured at DIV12 indicated by the small colored boxes. Scale bar, 5 µm. **b** Quantification of **a**, depicting median values (*N* = 3 experiments). Each data point represents one cell; Control (*n* = 50), *Tubb3* KD (*n* = 44). Statistics: Mann–Whitney Rank Sum Test. For graphical representation of the distribution, data are binned with a bin size of 0.2 a.u. **c** Live cell imaging of DIV 12 cultured hippocampal neurons cotransfected with KIF5C-Tomato-PEX and control or *Tubb3* knockdown constructs in axons. Image acquisition: 1 frame/s over 3 min. Scale bar, 20 µm. Motor movement in axonal segments in colored boxes of overview images (top panel) are visualized as kymographs (bottom panel). **d** Quantification of KIF5C-Tomato-PEX mobility in **a**, depicting median values (*N* = 3 experiments). *p < 0.05. Each data point represents one KIF5C signal; control (*n* = 41), knock down (*n* = 17). For graphical representation of the distribution, data are binned with a bin size of 0.1 µm/s. Statistics: Student’s *t* test. **e** Live cell imaging of DIV 12 cultured hippocampal neurons cotransfected with N-Cadherin-RFP and control or *Tubb3* knockdown constructs in axons. For details compare with **c**. **f** Quantification of N-Cadherin transport velocity in **e**, depicting median values (boxes) and mean values (black dot) (*N* = 3 experiments). *p < 0.05. Each data point represents one N-Cadherin signal; control (*n* = 21), knock down (*n* = 13). For graphical representation of the distribution, data are binned with a bin size of 0.1 µm/s. Statistics: Student's t-test

Remarkably, we found that tubulin polyglutamylation levels in neuronal axons were significantly reduced to 57% of control values following *Tubb3* knockdown (Fig. 8a, b). In subsequent time-lapse imaging experiments using living hippocampal neurons, we therefore analyzed the motility of the kinesin I-type motor protein KIF5C, which is highly mobile in axonal compartments [2]. A fluorescent fusion protein KIF5C-tomato-pex26, connected to a peroxisomal targeting signal [37], was used for neuronal live cell imaging. Under scrambled control conditions (Control), we observed a median value of KIF5C velocities of 1.31 µm/s (Fig. 8c, d). In contrast, the reduction of *Tubb3* gene expression levels (*Tubb3* KD) significantly increased the speed of movement to 1.47 µm/s (*p* = 0.018). Whereas the run length of the motor remained equal (Control: 24.0 µm; *Tubb3* KD: 23.9 µm; *p* = 0.838), the duration of movement turned out to be significantly reduced (Control: 29.0 s; *Tubb3* KD: 22.0 s; *p* = 0.017). In a further approach, we also performed time-lapse imaging with a fluorescent N-Cadherin fusion protein (mRFP-N-Cad) in axons [32]. N-Cadherin represents a physiological cargo of KIF5C motors in neurons, which head toward the synapse underlying the regulation of synaptogenesis and LTP [32, 34]. Similarly, as seen for KIF5C connected to peroxisomes, the velocity of axonal N-Cadherin movement significantly increased in neurons expressing reduced levels of the tubulin Tubb3 (median values, Control: 1.56 µm/s; *Tubb3* KD: 2.18 µm/s; *p* = 0.028) (Fig. 8e, f). Again, we observed that the run length of the motor remained equal (Control: 31.9 µm; *Tubb3* KD: 27.9 µm; *p* = 0.595), whereas the duration of movement turned out to be significantly reduced (Control: 31.0 s; *Tubb3* KD: 18.0 s; *p* < 0.001). We therefore conclude that the neuronal concentration of the tubulin isotype Tubb3 is a relevant parameter in the regulation of tubulin polyglutamylation and in the control of motor-cargo transport.

## Discussion

In this study, we report that microtubule growth and Tubb3 expression levels are sensitive to neuronal activity changes (Fig. 1). Our data further show that differential gene expression levels of individual tubulin isotypes translate into specific microtubule functions. We highlight the neuron-specific tubulin Tubb3 as an example to show that decreased Tubb3 levels alter microtubule growth parameters (Fig. 3) and microtubule-dependent transport of motor-cargo complexes (Fig. 8). Our study also reveals that decreased levels of a specific tubulin isotype (Tubb3) are partially compensated through Tubb4 upregulation (Fig. 4). Finally, we provide a computational Monte Carlo model that allows to simulate whether and how other tubulin isotypes affect microtubule growth velocities (Figs. 5 and 6).

It is presently incompletely understood how neuronal activity changes translate into changes of the microtubule cytoskeleton. A previous study identified that pharmacological activation of AMPARs or blockade of glycine receptors (GlyRs) alter tubulin polyglutamylation [54], however to our knowledge, cLTP-induced activity changes have not been studied in the context of microtubule growth or tubulin isotype expression. The observation that cLTP protocols induce increased levels of specific (Tubb1, Tubb3), but not all tubulin isotypes suggests for signaling cascades that mediate synapse-to-nucleus communication to target the expression of individual tubulin genes [57]. Tetraethylammonium (TEA)-induced cLTP has been shown to activate ERK-signaling in neurons, inducing phosphorylation of the nuclear transcription factor CREB [58]. Follow-up studies that search for signaling pathways connecting neuronal activity with tubulin expression could help to unravel the cooperation between both systems.

Previous in vitro microtubule dynamics assays had revealed that isolated microtubules display different growth rates, depending on the nature of individual tubulin isotypes. In fact, the growth velocity of Tuba1a/Tubb3 microtubules increased with decreasing amounts of Tubb3 [8]. Our data from hippocampal neurons (Fig. 3) confirm these in vitro results in a cellular context. Furthermore, it was reported that microtubules assemble faster in the presence of tau, as compared to the presence of MAP2 [10], suggesting that in addition to tubulin isotypes also microtubule-associated proteins contribute to their polymerization rates.

*Tubb3* knockdown in the present study led to a specific upregulation of Tubb4, but no other Tubb isotypes. These results differ from *Tubb3* knockout mice, which showed a general increase in other Tubbs in the range of 10–20% [30]. We conclude that these differences in the regulation of gene expression stem from the fact that our study did not manipulate the *Tubb3* gene but applied RNA silencing, which might be more physiological. Differences between gene knockout and knockdown studies have been reported in other cases and may be due to the position of the targeting vector or to the genetic background [59]. With respect to Tubb4, it should be noted that Tubb4 antibodies might have some cross-reactivities [7], which makes it difficult to fully exclude that other Tubbs remain unaffected. Likewise, the Tubb2 antibody detects Tubb2a and Tubb2b, wherefore some compensatory changes might have been missed. Although these technical limitations exist, modeling predicted a Tubb4 upregulation following Tubb3 knockdown (Fig. 6c), suggesting that the computational and experimental data in this study support each other.

Due to the number of tubulin genes and the large variety of possible dimer formations, our computational model can help to predict microtubule growth rates. This is not at least helpful because many antibodies against tubulins are not specific and show cross-reactivity [7]. Former microtubule models were based on a single dimer type, thereby neglecting different isotypes [40–43]. In contrast, the computational model in the present study incorporates three individual types of dimers, considering changes in isotype conformation. Since energy parameters were based on experimental EB3 data obtained from living cells, the model also considers the modulatory effects of microtubule binding proteins and posttranslational modifications in regulating microtubule growth velocities. Although it simplifies certain aspects of natural microtubules, it has been proven suitable to mimic experimental data (compare Figs. 3c, d, 6a). With that it provides a starting-point for future upgrades that include hydrolysis, catastrophe or shrinkage.

In general, the physiological connection between neuronal activity and the microtubule cytoskeleton is highly relevant, since activity changes not only alter cytoskeletal parameters, but microtubule-based transport in turn delivers mRNAs and proteins toward synapses to regulate synaptic transmission and/or plasticity. Increased microtubule growth rates (Figs. 1b, 3) might be beneficial for microtubules that invade into dendritic spines, following synaptic NMDA receptor activation [60, 61]. Since LTP triggers the KIF5-mediated delivery of AMPARs and N-Cadherin toward synapses, these molecules represent critical factors to monitor activity-dependent transport. Our data suggest that low Tubb3 levels accelerate their delivery (Fig. 8). Interestingly, exogenous Tubb3 expression in hippocampal neurons also led to increased MT growth rates in axons. Just as Tubb3 downregulation is compensated through increased Tubb4 expression, it is possible that increased Tubb3 expression would also affect other isotype levels. Remarkably, tubulin polyglutamylation levels, which are also known to regulate neuronal transport, are significantly decreased following Tubb3 knockdown (Fig. 8a, b), accompanied by increased transport rates. Consistent with these results, previous studies reported that increased polyglutamylation levels decrease the efficiency of neuronal transport whereas reduced polyglutamylation boosts axonal transport of mitochondria [36, 37, 56].

In summary, our study reveals that the concentration of Tubb3 matters, with respect to activity-dependent microtubule and transport functions. It provides a starting point to identify functions of other tubulin isotypes, using computational and experimental approaches.

## Supplementary Information

Below is the link to the electronic supplementary material.

Supplementary file1 (PDF 1486 KB)

## Data Availability

Further information and requests for resources and reagents should be directed to and will be fulfilled by the corresponding author.

## Code availability

Further information and requests for programing code should be directed to and will be fulfilled by the corresponding author.

## References

[1] Kapitein LC, Hoogenraad CC (2015). Building the neuronal microtubule cytoskeleton. Neuron.

[2] Hirokawa N, Niwa S, Tanaka Y (2010). Molecular motors in neurons: transport mechanisms and roles in brain function, development, and disease. Neuron.

[3] Akhmanova A, Steinmetz MO (2015). Control of microtubule organization and dynamics: two ends in the limelight. Nat Rev Mol Cell Biol.

[4] Janke C, Bulinski JC (2011). Post-translational regulation of the microtubule cytoskeleton: mechanisms and functions. Nat Rev Mol Cell Biol.

[5] Janke C, Kneussel M (2010). Tubulin post-translational modifications: encoding functions on the neuronal microtubule cytoskeleton. Trends Neurosci.

[6] Park JH, Roll-Mecak A (2018). The tubulin code in neuronal polarity. Curr Opin Neurobiol.

[7] Hausrat TJ, Radwitz J, Lombino FL, Breiden P, Kneussel M (2020). Alpha- and beta-tubulin isotypes are differentially expressed during brain development. Dev Neurobiol.

[8] Vemu A, Atherton J, Spector JO, Moores CA, Roll-Mecak A (2017). Tubulin isoform composition tunes microtubule dynamics. Mol Biol Cell.

[9] Panda D, Miller HP, Banerjee A, Luduena RF, Wilson L (1994). Microtubule dynamics in vitro are regulated by the tubulin isotype composition. Proc Natl Acad Sci USA.

[10] Banerjee A, Roach MC, Trcka P, Luduena RF (1990). Increased microtubule assembly in bovine brain tubulin lacking the type III isotype of beta-tubulin. J Biol Chem.

[11] Ti SC, Alushin GM, Kapoor TM (2018). Human beta-tubulin isotypes can regulate microtubule protofilament number and stability. Dev Cell.

[12] Pamula MC, Ti SC, Kapoor TM (2016). The structured core of human beta tubulin confers isotype-specific polymerization properties. J Cell Biol.

[13] Bliss TV, Lomo T (1973). Long-lasting potentiation of synaptic transmission in the dentate area of the anaesthetized rabbit following stimulation of the perforant path. J Physiol.

[14] Lu W, Man H, Ju W, Trimble WS, MacDonald JF, Wang YT (2001). Activation of synaptic NMDA receptors induces membrane insertion of new AMPA receptors and LTP in cultured hippocampal neurons. Neuron.

[15] Suzuki E, Okada T (2010). Group I metabotropic glutamate receptors are involved in TEA-induced long-term potentiation at mossy fiber-CA3 synapses in the rat hippocampus. Brain Res.

[16] Suzuki E, Okada T (2009). TEA-induced long-term potentiation at hippocampal mossy fiber-CA3 synapses: characteristics of its induction and expression. Brain Res.

[17] Kneussel M, Hausrat TJ (2016). Postsynaptic neurotransmitter receptor reserve pools for synaptic potentiation. Trends Neurosci.

[18] Makino H, Malinow R (2009). AMPA receptor incorporation into synapses during LTP: the role of lateral movement and exocytosis. Neuron.

[19] Bozdagi O, Shan W, Tanaka H, Benson DL, Huntley GW (2000). Increasing numbers of synaptic puncta during late-phase LTP: N-cadherin is synthesized, recruited to synaptic sites, and required for potentiation. Neuron.

[20] Ghiretti AE, Thies E, Tokito MK, Lin T, Ostap EM, Kneussel M (2016). Activity-dependent regulation of distinct transport and cytoskeletal remodeling functions of the dendritic kinesin KIF21B. Neuron.

[21] Guedes-Dias P, Holzbaur ELF (2019). Axonal transport: driving synaptic function. Science.

[22] Minoura I (2017). Towards an understanding of the isotype-specific functions of tubulin in neurons: technical advances in tubulin expression and purification. Neurosci Res.

[23] Tischfield MA, Baris HN, Wu C, Rudolph G, Van Maldergem L, He W (2010). Human TUBB3 mutations perturb microtubule dynamics, kinesin interactions, and axon guidance. Cell.

[24] Whitman MC, Andrews C, Chan WM, Tischfield MA, Stasheff SF, Brancati F (2016). Two unique TUBB3 mutations cause both CFEOM3 and malformations of cortical development. Am J Med Genet A.

[25] Lee MK, Tuttle JB, Rebhun LI, Cleveland DW, Frankfurter A (1990). The expression and posttranslational modification of a neuron-specific beta-tubulin isotype during chick embryogenesis. Cell Motil Cytoskeleton.

[26] Burgoyne RD, Cambray-Deakin MA, Lewis SA, Sarkar S, Cowan NJ (1988). Differential distribution of beta-tubulin isotypes in cerebellum. EMBO J.

[27] Saillour Y, Broix L, Bruel-Jungerman E, Lebrun N, Muraca G, Rucci J (2014). Beta tubulin isoforms are not interchangeable for rescuing impaired radial migration due to Tubb3 knockdown. Hum Mol Genet.

[28] Poirier K, Saillour Y, Bahi-Buisson N, Jaglin XH, Fallet-Bianco C, Nabbout R (2010). Mutations in the neuronal ss-tubulin subunit TUBB3 result in malformation of cortical development and neuronal migration defects. Hum Mol Genet.

[29] Xu X, Shangguan Y, Lu S, Wang W, Du C, Xiao F (2017). Tubulin beta-III modulates seizure activity in epilepsy. J Pathol.

[30] Latremoliere A, Cheng L, DeLisle M, Wu C, Chew S, Hutchinson EB (2018). Neuronal-specific TUBB3 is not required for normal neuronal function but is essential for timely axon regeneration. Cell Rep.

[31] Setou M, Seog DH, Tanaka Y, Kanai Y, Takei Y, Kawagishi M (2002). Glutamate-receptor-interacting protein GRIP1 directly steers kinesin to dendrites. Nature.

[32] Heisler FF, Lee HK, Gromova KV, Pechmann Y, Schurek B, Ruschkies L (2014). GRIP1 interlinks N-cadherin and AMPA receptors at vesicles to promote combined cargo transport into dendrites. Proc Natl Acad Sci USA.

[33] Bruses JL (2006). N-cadherin signaling in synapse formation and neuronal physiology. Mol Neurobiol.

[34] Mendez P, De Roo M, Poglia L, Klauser P, Muller D (2010). N-cadherin mediates plasticity-induced long-term spine stabilization. J Cell Biol.

[35] Huntley GW, Elste AM, Patil SB, Bozdagi O, Benson DL, Steward O (2012). Synaptic loss and retention of different classic cadherins with LTP-associated synaptic structural remodeling in vivo. Hippocampus.

[36] Magiera MM, Bodakuntla S, Ziak J, Lacomme S, Marques Sousa P, Leboucher S (2018). Excessive tubulin polyglutamylation causes neurodegeneration and perturbs neuronal transport. EMBO J.

[37] Lopes AT, Hausrat TJ, Heisler FF, Gromova KV, Lombino FL, Fischer T (2020). Spastin depletion increases tubulin polyglutamylation and impairs kinesin-mediated neuronal transport, leading to working and associative memory deficits. PLoS Biol.

[38] Kneussel M, Sanchez-Rodriguez N, Mischak M, Heisler FF (2021). Dynein and muskelin control myosin VI delivery towards the neuronal nucleus. iScience..

[39] Otmakhov N, Khibnik L, Otmakhova N, Carpenter S, Riahi S, Asrican B (2004). Forskolin-induced LTP in the CA1 hippocampal region is NMDA receptor dependent. J Neurophysiol.

[40] Gardner MK, Charlebois BD, Janosi IM, Howard J, Hunt AJ, Odde DJ (2011). Rapid microtubule self-assembly kinetics. Cell.

[41] VanBuren V, Odde DJ, Cassimeris L (2002). Estimates of lateral and longitudinal bond energies within the microtubule lattice. Proc Natl Acad Sci USA.

[42] Castle BT, McCubbin S, Prahl LS, Bernens JN, Sept D, Odde DJ (2017). Mechanisms of kinetic stabilization by the drugs paclitaxel and vinblastine. Mol Biol Cell.

[43] VanBuren V, Cassimeris L, Odde DJ (2005). Mechanochemical model of microtubule structure and self-assembly kinetics. Biophys J.

[44] Yue F, Cheng Y, Breschi A, Vierstra J, Wu W, Ryba T (2014). A comparative encyclopedia of DNA elements in the mouse genome. Nature.

[45] Nogales E, Whittaker M, Milligan RA, Downing KH (1999). High-resolution model of the microtubule. Cell.

[46] Gutierrez Y, Lopez-Garcia S, Lario A, Gutierrez-Eisman S, Delevoye C, Esteban JA (2021). KIF13A drives AMPA receptor synaptic delivery for long-term potentiation via endosomal remodeling. J Cell Biol.

[47] Hausrat TJ, Radwitz J, Lombino FL, Breiden P, Kneussel M (2021). Alpha- and beta-tubulin isotypes are differentially expressed during brain development. Dev Neurobiol.

[48] Aher A, Akhmanova A (2018). Tipping microtubule dynamics, one protofilament at a time. Curr Opin Cell Biol.

[49] Schapitz IU, Behrend B, Pechmann Y, Lappe-Siefke C, Kneussel SJ, Wallace KE (2010). Neuroligin 1 is dynamically exchanged at postsynaptic sites. J Neurosci.

[50] Yang C, Wu J, de Heus C, Grigoriev I, Liv N, Yao Y (2017). EB1 and EB3 regulate microtubule minus end organization and Golgi morphology. J Cell Biol.

[51] Kapitein LC, Yau KW, Hoogenraad CC (2010). Microtubule dynamics in dendritic spines. Methods Cell Biol.

[52] Kandel ER (2001). The molecular biology of memory storage: a dialogue between genes and synapses. Science.

[53] Park M, Penick EC, Edwards JG, Kauer JA, Ehlers MD (2004). Recycling endosomes supply AMPA receptors for LTP. Science.

[54] Maas C, Belgardt D, Lee HK, Heisler FF, Lappe-Siefke C, Magiera MM (2009). Synaptic activation modifies microtubules underlying transport of postsynaptic cargo. Proc Natl Acad Sci USA.

[55] Sirajuddin M, Rice LM, Vale RD (2014). Regulation of microtubule motors by tubulin isotypes and post-translational modifications. Nat Cell Biol.

[56] Bodakuntla S, Yuan X, Genova M, Gadadhar S, Leboucher S, Birling MC (2021). Distinct roles of alpha- and beta-tubulin polyglutamylation in controlling axonal transport and in neurodegeneration. EMBO J.

[57] Karpova A, Bar J, Kreutz MR (2012). Long-distance signaling from synapse to nucleus via protein messengers. Adv Exp Med Biol.

[58] Kanterewicz BI, Urban NN, McMahon DB, Norman ED, Giffen LJ, Favata MF (2000). The extracellular signal-regulated kinase cascade is required for NMDA receptor-independent LTP in area CA1 but not area CA3 of the hippocampus. J Neurosci.

[59] Hu X, Luo JH, Xu J (2015). The interplay between synaptic activity and neuroligin function in the CNS. Biomed Res Int.

[60] Hu X, Viesselmann C, Nam S, Merriam E, Dent EW (2008). Activity-dependent dynamic microtubule invasion of dendritic spines. J Neurosci.

[61] Jaworski J, Kapitein LC, Gouveia SM, Dortland BR, Wulf PS, Grigoriev I (2009). Dynamic microtubules regulate dendritic spine morphology and synaptic plasticity. Neuron.

