# Supplementary material for: Tubb3 expression levels are sensitive to neuronal activity changes and determine microtubule growth and kinesin-mediated transport
Source: Cell Mol Life Sci. 2022 Oct 29;79(11):575. doi: 10.1007/s00018-022-04607-5 (PMC9617967; doi:10.1007/s00018-022-04607-5)
Supplement: Supplementary file 1 — Supplementary file1 (PDF 1486 KB) [file 18_2022_4607_MOESM1_ESM.pdf]

SUPPLEMENTARY INFORMATION

Tubb3 expression levels are sensitive to neuronal activity changes and determine microtubule growth and kinesin-mediated transport

Cellular and Molecular Life Sciences

Jennifer Radwitz[1], Torben J. Hausrat[1], Frank F. Heisler[1], Philipp C. Janiesch[1], Yvonne Pechmann[1] Michael Rübhausen[2], Matthias Kneussel[1,3].

[1]Institute for Molecular Neurogenetics, Center for Molecular Neurobiology, ZMNH, University Medical Center Hamburg-Eppendorf, Falkenried 94, 20251 Hamburg, Germany

[2]Institut für Nanostruktur- und Festkörperphysik, Center for Free Electron Laser Science (CFEL), University of Hamburg, Luruper Chaussee 149, 22761 Hamburg, Germany

[3]Correspondence: matthias.kneussel@zmnh.uni-hamburg.de

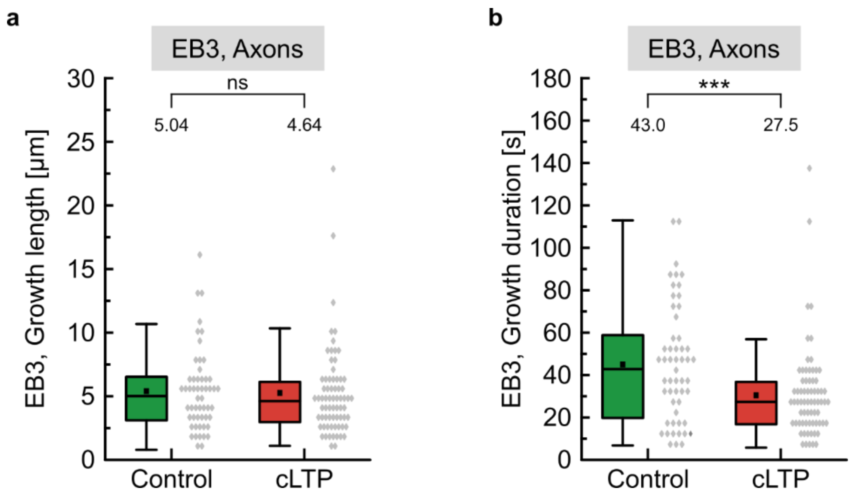

Figure S1. Supplemental Data to Figure 1a, b.

Quantification of microtubule growth lengths and growth durations in axons, depicting median values (N=3 experiments). *** $p<0.001$, ns: not significant. Each data point represents one EB3 comet; control (n=54), cLTP (n=70). For graphical representation of the distribution, data are binned with a bin size of (a) 0.75 µm and (b) 5 s. Statistics: Mann-Whitney Rank Sum Test.

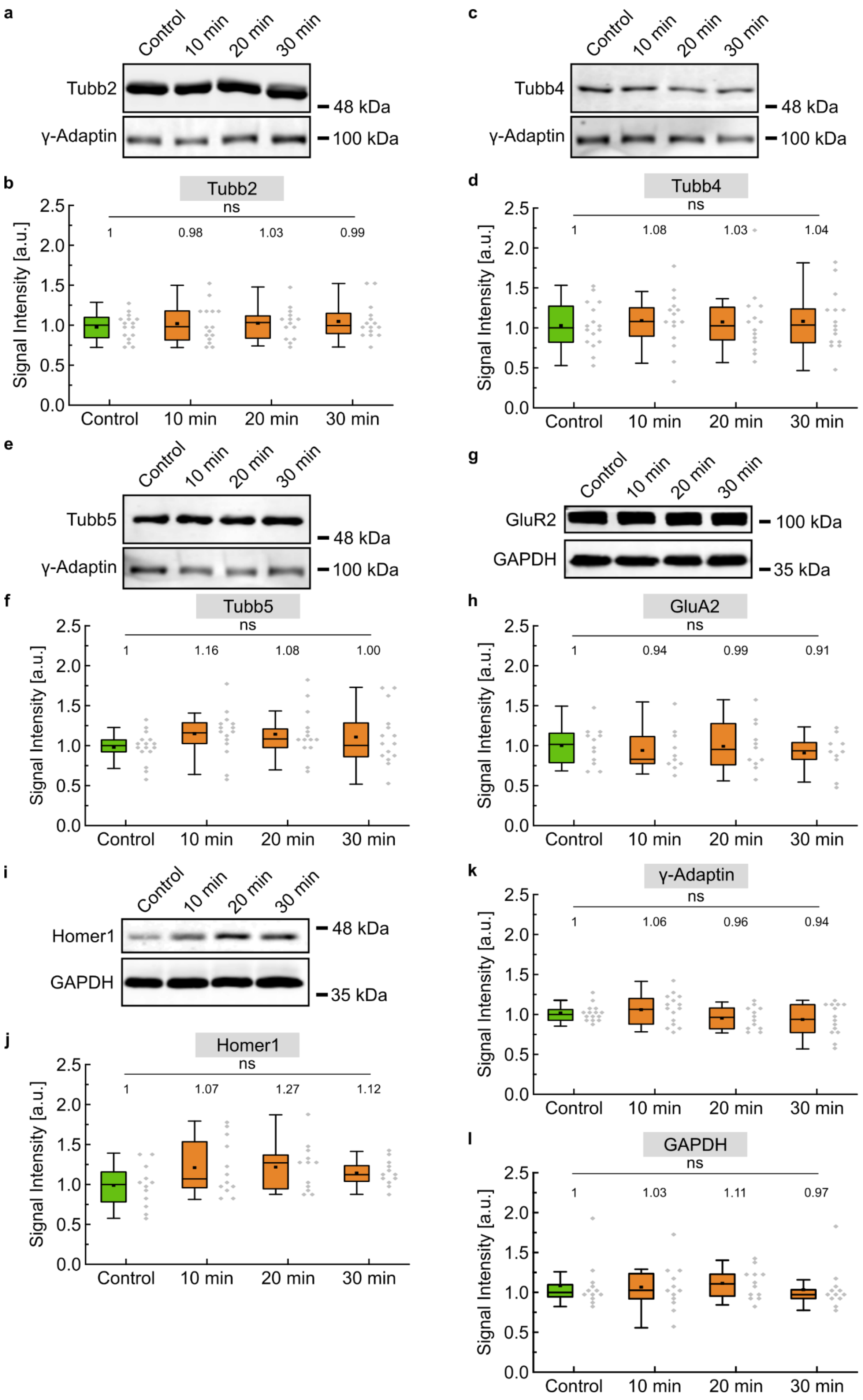
a
Control
10 min
20 min
30 min
Tubb2
γ-Adaptin
48 kDa
100 kDa
b
Tubb2
ns
1
0.98
1.03
0.99
Signal Intensity [a.u.]
2.5
2.0
1.5
1.0
0.5
0.0
Control
10 min
20 min
30 min
c
Control
10 min
20 min
30 min
Tubb4
γ-Adaptin
48 kDa
100 kDa
d
Tubb4
ns
1
1.08
1.03
1.04
Signal Intensity [a.u.]
Control
10 min
20 min
30 min
e
Control
10 min
20 min
30 min
Tubb5
γ-Adaptin
48 kDa
100 kDa
f
Tubb5
ns
1
1.16
1.08
1.00
Signal Intensity [a.u.]
Control
10 min
20 min
30 min
g
Control
10 min
20 min
30 min
GluR2
GAPDH
100 kDa
35 kDa
h
GluA2
ns
1
0.94
0.99
0.91
Signal Intensity [a.u.]
Control
10 min
20 min
30 min
i
Control
10 min
20 min
30 min
Homer1
GAPDH
48 kDa
35 kDa
j
Homer1
ns
1
1.07
1.27
1.12
Signal Intensity [a.u.]
Control
10 min
20 min
30 min
k
γ-Adaptin
ns
1
1.06
0.96
0.94
Signal Intensity [a.u.]
Control
10 min
20 min
30 min
l
GAPDH
ns
1
1.03
1.11
0.97
Signal Intensity [a.u.]
Control
10 min
20 min
30 min

Figure S2. Supplemental Data to Figure 1c-j.

Western blot analysis and quantification of tubulin isotype expression in acute hippocampal slices, following chemical induction of long-term potentiation (cLTP). (a, b) Tubb2, (c, d) Tubb4, (e, f) Tubb5, (g, h) GluA2, (i, j) Homer1, (k) gamma-Adaptin, (l) GAPDH. Median values are depicted. Black dots represent mean values. For graphical representation of the distribution, data were binned with a bin size of 0.05 a.u. Statistics: One way ANOVA. (ns: not significant).

a

b

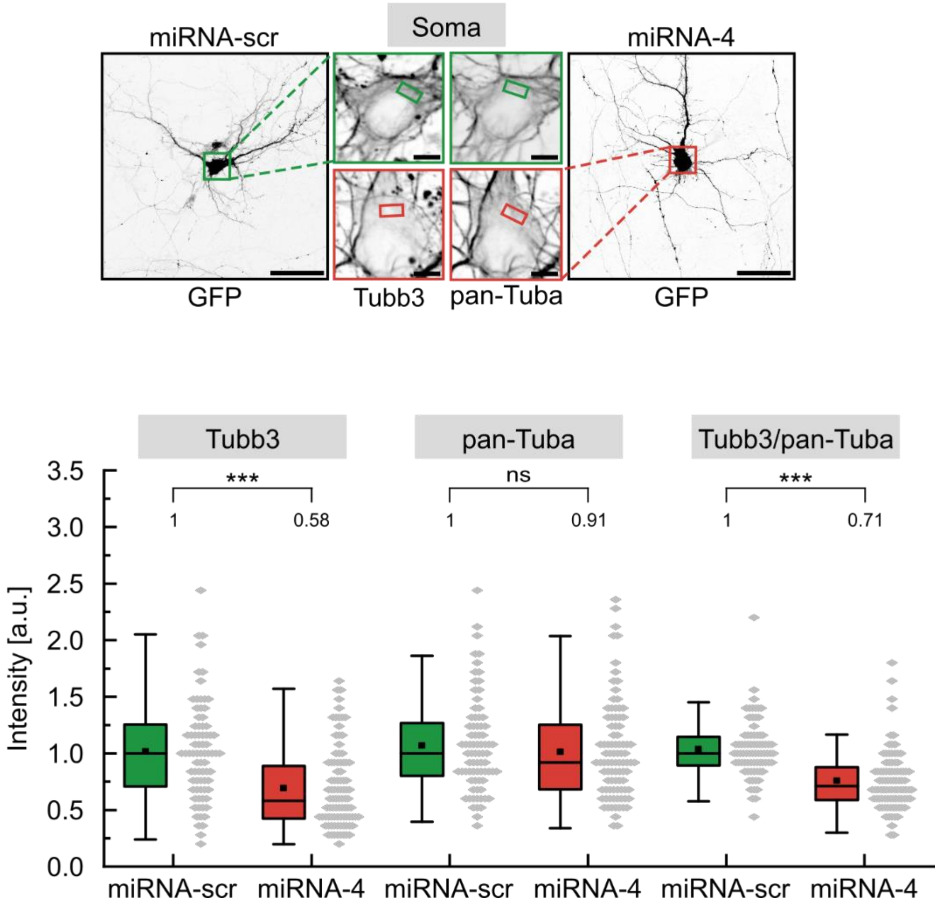

Figure S3. Supplemental Data to Figure 2.

(a) Cultured hippocampal neurons transfected with miRNA-scr or miRNA-4, respectively. Scale bar, 50 µm. Somata in boxed regions are enlarged in the middle (green: control, red: knockdown). Tubb3 and pan-Tuba intensities were measured at DIV12 inside the soma, indicated by the small colored boxes. Scale bar, 5 µm. (b) Quantification of a, depicting median values (N=3 experiments). On the right Tubb3 data were normalized to pan-Tuba. Each data point represents one cell; miRNA-scr (n = 78), miRNA-4 (n = 91). Statistics: Mann-Whitney Rank Sum Test. For graphical representation of the distribution, data are binned with a bin size of 0.08 a.u.

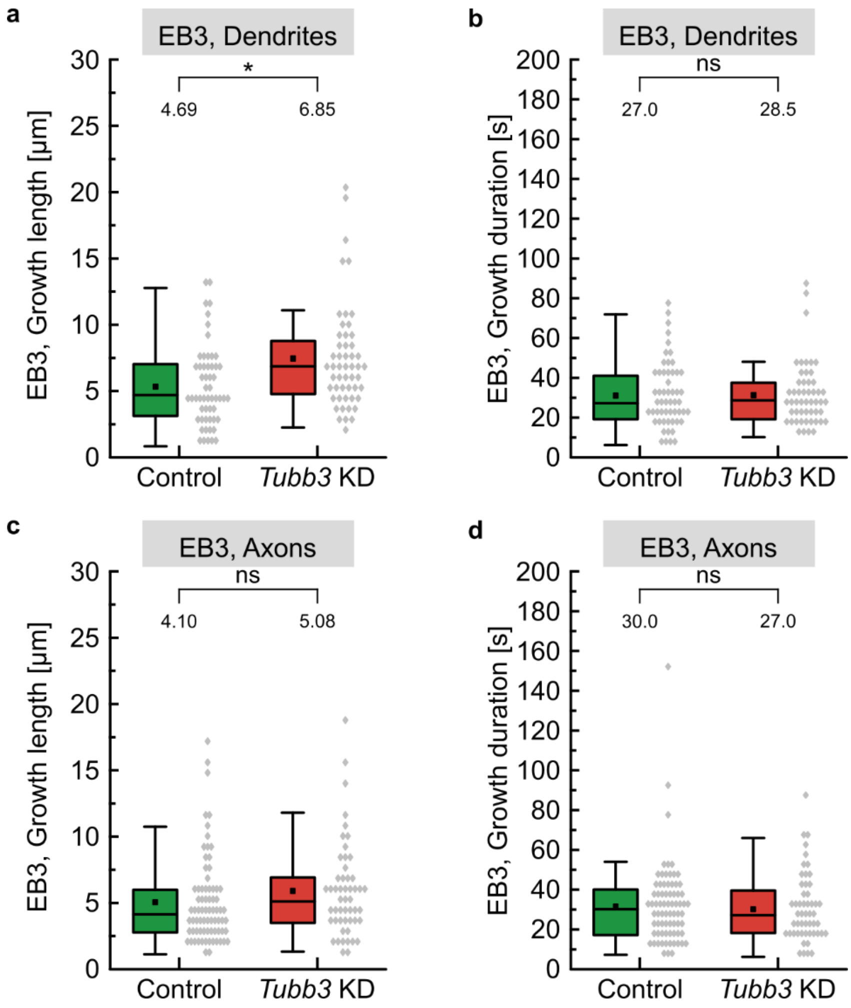

Figure S4. Supplemental Data to Figure 3.

(a, b) Quantification of microtubule growth lengths and growth durations in dendrites, depicting median values (N=3 experiments). * $p<0.05$, ns: not significant. Each data point represents one EB3 comet; control (n=55), knockdown (n=52). For graphical representation of the distribution, data are binned with a bin size of (a) 0.8 µm and (b) 5s. (c, d) Quantification of microtubule growth lengths and growth durations in axons depicting median values (N=3 experiments). ns: not significant. Each data point represents one EB3 comet; control (n=75), knockdown (n=52). For graphical representation of the distribution, data are binned with a bin size of (c) 0.8 µm and (d) 5 s. Statistics: Mann-Whitney Rank Sum Test.

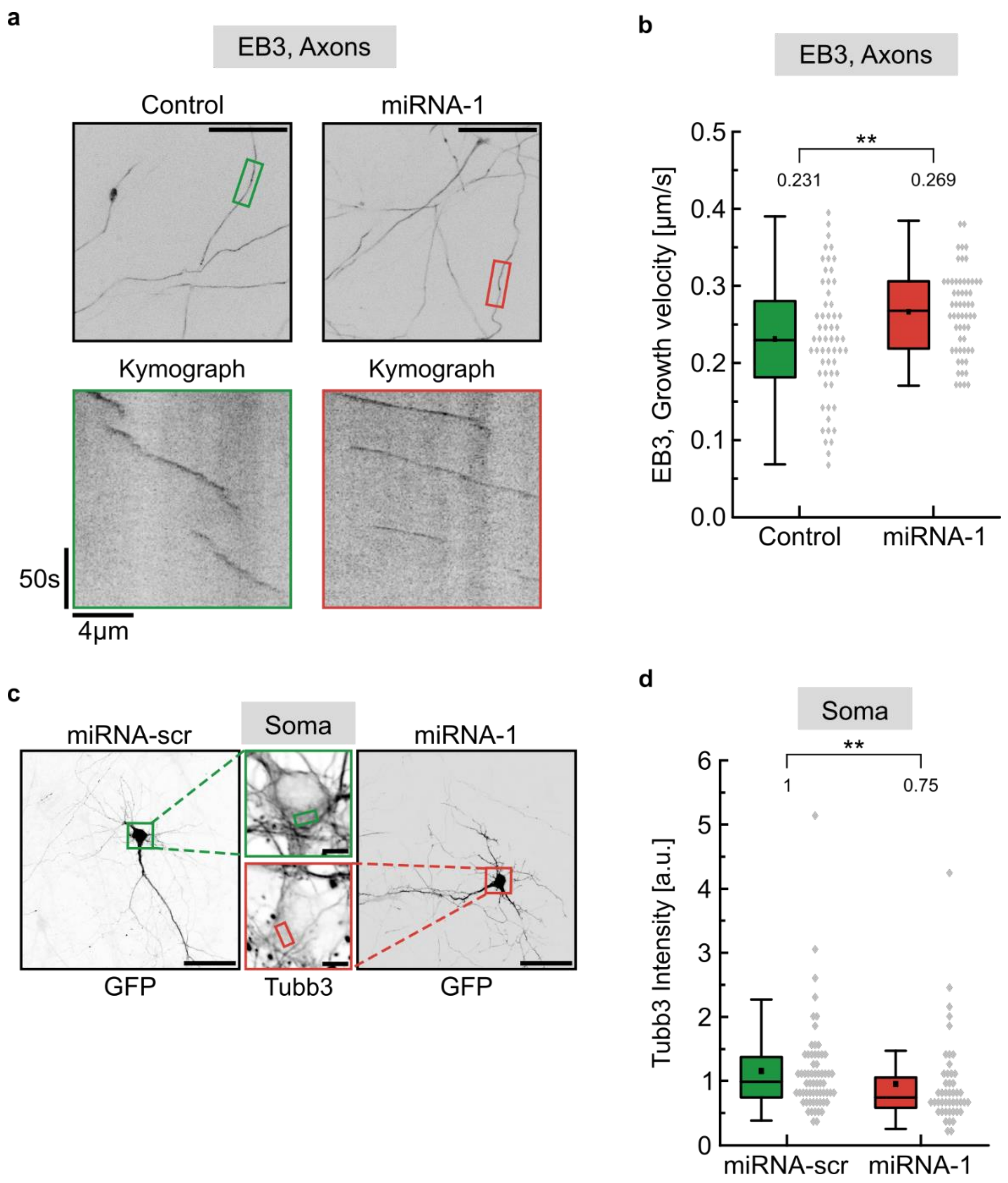

Figure S5. Supplemental Data to Figure 3.

(a) Live cell imaging of DIV 12 cultured hippocampal neurons cotransfected with EB3-Tomato and control or *Tubb3* knockdown construct (miRNA-1) in axons. Image acquisition: 1 frame/s over 3 min. Scale bar, 20 µm. EB3 comets in axonal segments in colored boxes of overview images (top panel) are visualized by kymographs (bottom panel). (b) Quantification of a, depicting median values (N=3 experiments). ** $p<0.01$. Each data point represents one EB3 comet; control (n=58), knockdown (n=58). For graphical representation of the distribution, data are binned with a bin size of 0.015 µm/s. Statistics: Welch's t-test. (c) Cultured hippocampal neurons transfected with miRNA-scr or miRNA-1, respectively. Scale bar, 50 µm. Somata in boxed regions are enlarged in the middle (green: control, red: knockdown). Tubb3 intensities were measured at DIV12 inside the soma, indicated by the small colored boxes. Scale bar, 5 µm. (d) Quantification of c, depicting median values (N=2 experiments). Each

data point represents one cell; miRNA-scr (n = 63), miRNA-1 (n = 46). Statistics: Mann-Whitney Rank Sum Test. For graphical representation of the distribution, data are binned with a bin size of 0.15 a.u.

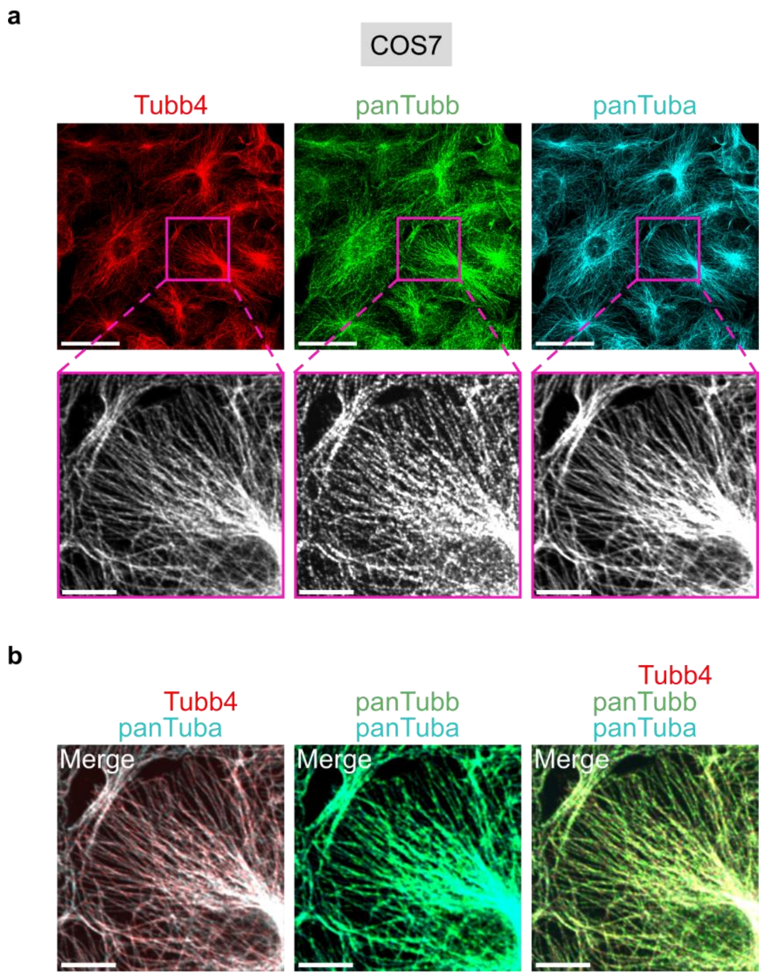

Figure S6. Supplemental Data to Figure 4.

(a) Cultured COS7 cells immunostained with Tubb4-, panTubb- and panTuba-specific antibodies. Scale bar 40 µm. Enlargements of the boxed regions depict filamentous tubulin. Scale bar 10 µm. (b) Merged images of enlarged regions of a. Scale bar 10 µm.

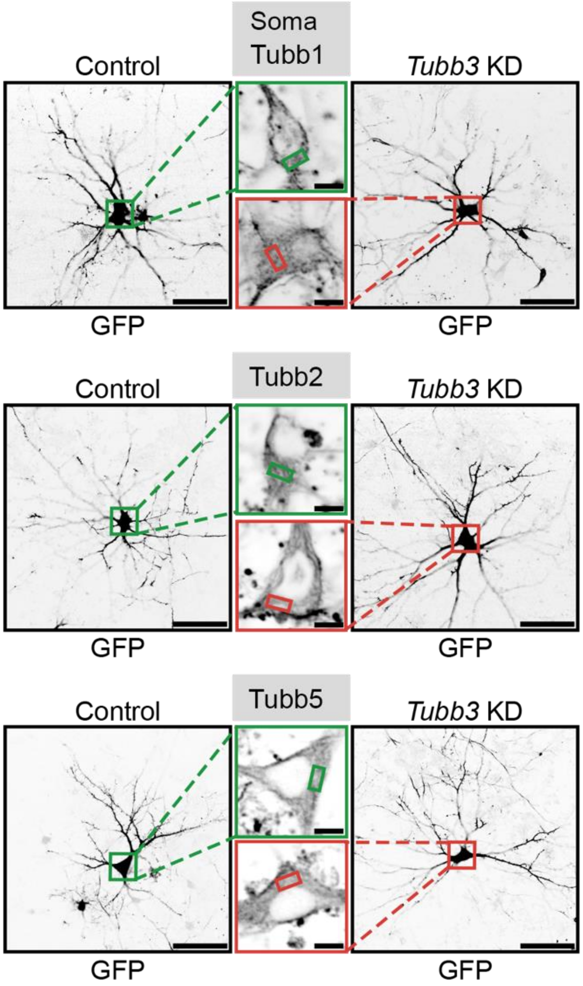

Figure S7. Supplemental Data to Figure 4.

Cultured hippocampal neurons (DIV12) transfected with control or *Tubb3* knockdown constructs. From top down: Detection with a specific Tubb1, Tubb2 and Tubb5 antibody. Scale bar, 50 µm. Middle panels: Enlargements of cell somata boxed regions. Tubulin intensities were measured inside the soma, indicated by the small colored boxes. DAPI staining of the nucleus (not shown) was used to verify that tubulin intensity measurements were performed in the cytoplasm. Scale bar, 5 µm.

G_{long}(Tubb3) in RT	G_{lat}(Tubb3) in RT	G_{long}(Tubb4) in RT	G_{lat}(Tubb4) in RT	velocity control in µm/s	velocity knockdown in µm/s
-9	-1.5	-7	-6.5	0.165	0.204
-9	-0.5	-7	-6.5	0.162	0.207
-8.5	-6.5	-6	-5.5	0.168	0.201
-8.5	-3	-8	-4.5	0.168	0.203
-8.5	-3	-7	-5.5	0.171	0.207
-8.5	-2.5	-6.5	-6	0.169	0.199
-8.5	-2	-7.5	-5	0.164	0.198
-8	-5.5	-6.5	-5.5	0.167	0.199
-8	-2	-8	-5.5	0.171	0.199
-8	-1.5	-7.5	-6	0.173	0.200
-7.5	-7	-6	-6	0.165	0.204
-7.5	-6	-6.5	-6	0.164	0.202
-7.5	-3	-8.5	-4	0.172	0.200
-7.5	-1.5	-8	-4.5	0.168	0.198
-7.5	-1	-7.5	-7	0.171	0.201
-7.5	-1	-7.5	-6.5	0.163	0.204
-7.5	-1	-7.5	-5	0.162	0.209
-7	-6.5	-7.5	-4.5	0.162	0.198
-7	-5	-7	-6	0.173	0.206
-7	-3.5	-9	-3	0.172	0.197
-7	-2.5	-7	-6.5	0.166	0.209
-7	-1.5	-7.5	-6	0.164	0.196
-7	-1	-8	-6	0.173	0.197
-7	-1	-8	-5	0.169	0.197
-7	-1	-7	-7	0.162	0.198
-6.5	-5.5	-7	-6	0.170	0.204
-6.5	-5.5	-7	-5.5	0.172	0.197
-6.5	-5	-7	-6.5	0.169	0.197
-6.5	-4.5	-8.5	-4	0.164	0.202
-6.5	-4.5	-8.5	-3.5	0.169	0.203
-6.5	-4.5	-7.5	-5	0.164	0.195
-6.5	-4	-7	-7	0.170	0.194
-6.5	-3.5	-7.5	-5	0.165	0.202
-6.5	-2	-7.5	-6	0.167	0.197
-6.5	-1	-8.5	-4.5	0.171	0.208
-6.5	-1	-8	-5	0.167	0.202
-6	-4.5	-9	-4	0.168	0.196
-6	-3.5	-8.5	-4	0.169	0.196
-6	-3.5	-8	-5.5	0.166	0.202
-6	-3.5	-7	-5	0.165	0.196
-6	-2.5	-8.5	-4	0.164	0.204
-6	-2.5	-8	-6	0.167	0.205
-6	-2.5	-7.5	-6	0.173	0.205
-6	-2	-9	-4	0.166	0.206

-6	-2	-8	-4.5	0.162	0.208
-6	-1.5	-7.5	-6	0.172	0.202
-6	-1	-8.5	-4.5	0.166	0.194
-6	-1	-7	-7	0.162	0.194
-5.5	-4.5	-8.5	-4	0.168	0.201
-5.5	-4.5	-7.5	-5	0.169	0.195
-5.5	-3	-8.5	-4.5	0.166	0.202
-5.5	-2.5	-7.5	-7	0.167	0.207
-5.5	-2.5	-7	-7	0.161	0.206
-5.5	-1	-7.5	-7	0.161	0.198
-5	-4.5	-8.5	-4.5	0.168	0.206
-5	-4.5	-8.5	-4	0.166	0.206
-5	-4	-7.5	-5	0.165	0.195
-5	-3.5	-8.5	-4.5	0.172	0.206
-5	-3.5	-7.5	-7.5	0.165	0.208
-5	-3	-8	-5.5	0.163	0.198
-5	-3	-7.5	-6	0.172	0.205
-5	-2.5	-8	-6	0.172	0.195
-5	-2	-7	-7	0.168	0.195
-5	-1.5	-7.5	-7.5	0.167	0.208
-5	-1	-8.5	-4.5	0.172	0.197
-5	-1	-8	-6	0.164	0.193
-4.5	-4.5	-7	-7	0.162	0.193
-4.5	-4	-7.5	-6.5	0.168	0.202
-4.5	-3	-8.5	-4.5	0.168	0.206
-4.5	-3	-8	-5	0.169	0.198
-4.5	-1	-8	-4.5	0.168	0.199
-4	-3.5	-8	-5.5	0.165	0.194
-4	-3	-8	-5.5	0.165	0.201
-4	-3	-7.5	-6.5	0.172	0.194
-4	-2.5	-8	-5	0.172	0.199
-4	-1.5	-7	-6.5	0.162	0.204
-4	-1	-8	-6	0.167	0.205
-4	-1	-7.5	-7	0.163	0.195
-3.5	-3.5	-7.5	-6.5	0.172	0.200
-3.5	-3	-8.5	-4.5	0.167	0.193
-3.5	-3	-8.5	-4	0.162	0.199
-3.5	-2.5	-9	-3.5	0.169	0.198
-3.5	-2	-8	-4.5	0.170	0.194
-3.5	-2	-7.5	-7.5	0.165	0.208
-3	-3	-9	-4	0.169	0.205
-3	-2.5	-8	-7	0.167	0.203
-3	-1.5	-8.5	-5	0.168	0.198
-3	-1.5	-8	-8	0.165	0.197

Table S1. Energy parameter settings - Supplemental Data to Figure 5.

For each energy parameter set, one growth velocity value was calculated under control or *Tubb3* knockdown conditions, respectively. Experimental mean values of microtubule growth in axons (compare with Figure 3d) were 0.167±0.006 µm/s (control) and 0.201±0.008 µm/s (*Tubb3* knockdown). Energy parameter sets that generated velocities in these ranges were selected. Parameter sets that coincided for control and knockdown conditions were filtered. This resulted in 88 parameter settings that fulfilled the requirements.
